# The liquid fraction from hydrothermal pretreatment of wheat straw provides lytic polysaccharide monooxygenases with both electrons and H_2_O_2_ co-substrate

**DOI:** 10.1186/s13068-019-1578-5

**Published:** 2019-10-08

**Authors:** Riin Kont, Ville Pihlajaniemi, Anna S. Borisova, Nina Aro, Kaisa Marjamaa, Judith Loogen, Jochen Büchs, Vincent G. H. Eijsink, Kristiina Kruus, Priit Väljamäe

**Affiliations:** 1 Institute of Molecular and Cell Biology, University of Tartu, Tartu, Estonia; 2 VTT Technical Research Centre of Finland Ltd, Espoo, Finland; 3 Department of Biochemical Engineering (AVT.BioVT), RWTH Aachen University, Aachen, Germany; 4 Norwegian University of Life Sciences (NMBU), Ås, Norway

**Keywords:** Lytic polysaccharide monooxygenase, Hydrogen peroxide, Hydrothermal pretreatment, Wheat straw, Phenolic compounds, Oxidation, Cellulose, Chitin, *Trichoderma reesei* LPMO9A, *Serratia marcescens* LPMO10A

## Abstract

**Background:**

Enzyme-aided valorization of lignocellulose represents a green and sustainable alternative to the traditional chemical industry. The recently discovered lytic polysaccharide monooxygenases (LPMOs) are important components of the state-of-the art enzyme cocktails for cellulose conversion. Yet, these monocopper enzymes are poorly characterized in terms of their kinetics, as exemplified by the growing evidence for that H_2_O_2_ may be a more efficient co-substrate for LPMOs than O_2_. LPMOs need external electron donors and one key question of relevance for bioprocess development is whether the required reducing power may be provided by the lignocellulosic substrate.

**Results:**

Here, we show that the liquid fraction (LF) resulting from hydrothermal pretreatment of wheat straw supports LPMO activity on both chitin and cellulose. The initial, transient activity burst of the LPMO reaction was caused by the H_2_O_2_ present in the LF before addition of LPMO, while the steady-state rate of LPMO reaction was limited by the LPMO-independent production of H_2_O_2_ in the LF. H_2_O_2_ is an intermediate of LF oxidation as evidenced by a slow H_2_O_2_ accumulation in LF, despite high H_2_O_2_ production rates. This H_2_O_2_ scavenging ability of LF is important since high concentrations of H_2_O_2_ may lead to irreversible inactivation of LPMOs.

**Conclusions:**

Our results support the growing understanding that fine-tuned control over the rates of H_2_O_2_ production and consumption in different, enzymatic and non-enzymatic reactions is essential for harnessing the full catalytic potential of LPMOs in lignocellulose valorization.

## Background

Lignocellulosic biomass is the most abundant source of renewable carbon in Nature. Its enzyme-aided valorization to biofuels and building blocks for the chemical industry provides a green and sustainable alternative to the petroleum-based chemistry. Because of its inherent recalcitrance, the lignocellulose of plant cell walls requires mechano-chemical pretreatment to increase its susceptibility to enzymatic conversion. Hydrothermal pretreatment does not require use of chemicals and is a simple and environment friendly method that has proven to be efficient for different biomasses [1]. The most abundant soluble by-products from hydrothermal pretreatment, and from analogous dilute acid pretreatment [1], are hemicellulose-derived mono- and oligosaccharides, and various phenolic compounds [2–4].

The enzymatic deconstruction of the polysaccharides, i.e., mainly cellulose, in pretreated lignocellulosic biomass relies on synergistic interplay between enzymes with different substrate specificities and modes of action. An important breakthrough in the field came in 2010 when it was discovered that a chitin-binding protein of *Serratia marcescens* (CBP21) is an enzyme that catalyzes oxidative cleavage of glycosidic bonds [5]. Today, these enzymes are referred to as lytic polysaccharide monooxygenases (LPMO). Since then, LPMOs with different substrate specificities have been discovered, in most kingdoms of life and it is well reported that LPMOs synergize with conventional glycoside hydrolases and improve the saccharification of biomass [6–23]. Nowadays, the LPMOs are important components of the state-of-the-art enzyme cocktails used in industrial degradation of lignocellulosic biomass [24].

LPMOs are monocopper enzymes with a flat binding surface that enables binding to the ordered crystalline regions of substrate [25]. They catalyze oxidative cleavage of glycosidic bonds by hydroxylating, either the C1 or the C4 of the scissile bond [26], resulting in the formation of a lactone or ketone, respectively [27]. According to the originally proposed mechanism [5], LPMOs require O_2_ as a co-substrate and delivery of two electrons from an external electron donor per glycosidic bond cleavage [27, 28]. A number of different compounds can support LPMOs with electrons [29], including phenolic compounds [7, 30–35] or lignin and its fragments [7, 31, 36–38], which are both expected to be present in liquid fractions emerging during thermochemical pretreatment of biomass [3, 39–45].

Another breakthrough in the LPMO field was made in 2017 when it was shown that LPMOs can use H_2_O_2_ instead of O_2_ [46]. Although the nature of the true co-substrate of LPMOs is a matter of scientific debate, the fact is that H_2_O_2_ is used much more efficiently than O_2_ [46–49]. The H_2_O_2_-based mechanism also depends on the presence of external electron donor, but here the reductant is only needed for the initial “priming” of the Cu(II) resting state of the LPMO to its catalytically active Cu(I) form [46, 50]. Once in its active form, an LPMO can catalyze a number of oxidative cleavages until the active site copper happens to be re-oxidized, either by H_2_O_2_ or O_2_ [50]. The reductants that are required for LPMO activation are amenable to abiotic oxidation by O_2_ and H_2_O_2_ is often a product of these oxidations, complicating experimental assessment of LPMO action [51]. LPMOs are also a subject of irreversible inactivation by non-productive redox processes in the catalytic center [46, 48, 49, 51]. Therefore, the fine-tuned control over the concentration of the oxygen co-substrate is of utmost importance in harnessing the full catalytic potential of LPMOs.

Here, we have studied to what extend liquid fractions (LFs) from hydrothermal pre-treatment of wheat straw support the degradation of cellulose by a *Trichoderma reesei* LPMO (*Tr*LPMO9A) as well as the degradation of chitin by a *Serratia marcescens* LPMO (*Sm*LPMO10A). Reducing power and LPMO-independent generation of H_2_O_2_ in such liquid fractions were found to drive the LPMO activity, shedding new light on the possible interplay between biomass pretreatment and subsequent saccharification by LPMO-containing enzyme cocktails.

## Methods

### Substrates and reagents

^14^C-labeled chitin nanowhiskers (CNWs) were prepared by *N*-acetylation of non-labeled CNWs with ^14^C-acetic anhydride exactly as described in Kuusk et al. [52]. ^14^C-labeled bacterial cellulose was prepared by laboratory fermentation of *Gluconobacter xylinum* (ATCC 53582) in a medium supplied with uniformly ^14^C-labeled glucose as described before [53, 54]. ^14^C-labeled microcrystalline cellulose (BMCC) was prepared by incubating ^14^C-labeled bacterial cellulose (2 g L^−1^) with 1.0-M HCl at 100 °C for 3 h followed by extensive washing with water. The specific radioactivities of CNWs and BMCC were 4.18 × 10^6^ and 6.4 × 10^5^ dpm mg^−1^, respectively. Before using in experiments, both CNWs and BMCC were treated with EDTA to remove divalent metal ions. For that the polysaccharide (2 g L^−1^) was incubated with 10-mM EDTA at room temperature overnight. The EDTA-treated polysaccharides were extensively washed with water and 50-mM sodium acetate (pH 5.0) through repetitive centrifugation and re-suspension steps. The water was Milli-Q ultrapure water that had been passed through a column with Chelex^®^ 100 resin (BioRad). The stock solution of sodium acetate buffer was stored over beads of Chelex^®^ 100 resin. Solutions of H_2_O_2_ and ascorbic acid were made freshly before use.

### Enzymes

*Sm*LPMO10A was produced and purified as described before [55]. *Tr*LPMO9A was produced as follows: the gene encoding *Tr*LPMO9A was obtained by PCR from genomic DNA of *T. reesei* QM9414 using oligonucleotides SO1 (5′AACCCAATAGTCAACCGCGGACTGCGCATCATGATCCAGAAGCTTTCCAA) and SO2 (5′ACCGGTGCGTCAGGCTTTCGCCACGGAGCTCTAGTTAAGGCACTGGGCGT). The expression vector was assembled with the yeast recombination cloning method using the PCR fragment and *Pac*I (Fermentas) linearized pTTv248 vector backbone [56]. The final expression vector contained a targeting sequence for the *cbh1* locus (tre123989), 2184 bp of *cbh1* 5′ region containing the *cbh1* promoter and 1745 bp of *cbh1* 3′ region and the hphR selection marker [57, 58]. After plasmid rescue and transformation into *E. coli* [56], the construct was verified by sequencing. The expression cassette was liberated from the vector backbone with *Pme*I (Fermentas) restriction enzyme digestion prior to transformation. *T. reesei* strain M362 (M124 Δtre72567, Δtre122081 and Δtre120312), which is deleted for three major cellulase genes (*cbh2*, *egl1*, *egl2*), was transformed with the expression cassette and grown on MM + hygromycin transformation plates [59]. Transformants were screened first by PCR for 5′ and 3′ flank integration into the *cbh1* locus and absence of the open reading frame for *cbh1* (for PCR primers see Additional file 1: Table S1). The generated strain, M1906, was cultivated for protein production in a BioFlo 510 15L reactor (New Brunswick Scientific, USA) with 10-L operating volume, using a culture medium containing lactose (40 g L^−1^), spent grain extract (30 g L^−1^) (Harbro Ltd, UK), KH_2_PO_4_ (5 g L^−1^), (NH_4_)_2_SO_4_ (5 g L^−1^) MgSO_4_ (2.4 mM), CaCl_2_ (4.1 mM), CoCI_2_ (3.7 mg L^−1^), FeSO_4_·7H_2_O (5 mg L^−1^), ZnSO_4_·7H_2_O (1.4 mg L^−1^) and MnSO_4_·7H_2_O (1.6 mg L^−1^) and Struktol J647 Antifoam (1 mL L^−1^). The cultivation was carried out at 28 °C and pH 4.8–4.9. The pH was controlled by addition of base (5% NH_4_OH) or acid (10% H_3_PO_4_) when necessary. The cultivation was done with constant aeration (10 L min^−1^) and mixing (150–500 rpm) was adjusted to keep the oxygen concentration at 30%. Lactose (20% (w/v)) feeding was initiated after 61-h cultivation and adjusted as described in [60]. The cultivation was terminated after 163 h. The culture supernatant was concentrated with Millipore Pellicon 2 filter (10 kDa membrane cutoff). *Tr*LPMO9A was purified with the following procedure: 0.5 L of the concentrated culture supernatant was exchanged to 10-mM sodium phosphate pH 7.0 using a Sephadex G25 column (column volume 3.5 L), applied to a DEAE Sepharose anion exchange column (column volume 1.0 L) and eluted using a 0–100 mM (30 column volumes) NaCl gradient. The fractions were analyzed with SDS-PAGE using 4–20% Stain-Free gradient gels and Imaging System (BioRad, Hercules, California, USA). The fractions containing *Tr*LPMO9A were pooled and buffer was exchanged to 50 mM sodium acetate pH 5 using ultrafiltration (Prep/Scale-TFF 1ft^2^Cartridge, PTGC 10 k Polyethersulfone). Ammonium sulfate was added to the pooled sample to a final concentration 0.5 M after which the sample was applied to a Phenyl-Sepharose HIC column (column volume 0.14 L). *Tr*LPMO9A was collected from the flow-through. The buffer of purified *Tr*LPMO9A was changed to 25-mM sodium acetate pH 5.0 and the enzyme was concentrated using ultrafiltration as described above. SDS-PAGE analysis of purified *Tr*LPMO9A is shown in the Additional file 1: Fig. S1. Contaminating endoglucanase [61], xylanase [62] and mannanase trace activities [63] of purified *Tr*LPMO9A were 1.2, 3.2, and 2.6 nkat mg^−1^ protein, respectively.

The purified LPMOs (around 150 µM) were copper saturated by overnight incubation with CuSO_4_ (threefold molar excess) and subsequent removal of free copper by ultrafiltration. The concentration of LPMOs was determined by measuring absorbance at 280 nm using molar extinction coefficients of 35,200 and 54,360 M^−1^ cm^−1^ for *Sm*LPMO10A and *Tr*LPMO9A, respectively. Horseradish peroxidase (HRP, Sigma) was used as purchased. The concentration of HRP was determined by measuring absorbance at 403 nm using molar extinction coefficient of 102,000 M^−1^ cm^−1^.

### Hydrothermal pretreatment of wheat straw

Chopped wheat straw from Finland was pre-soaked with water to 50% dry matter content and loaded into a 30-L pressure reactor with a batch size of 1.71-kg dry matter. The material was heated to 195 °C with direct steam injection and a pressurized water jacket, and the temperature was maintained for 15 min. After the treatment, the material was quickly cooled to 80 °C with the water jacket, and dissolved material was extracted by pumping 80 °C water through the material bed. The first 6 L of extract was collected and this material is hereafter referred to as liquid fraction (LF). The pretreated solids were collected manually. During the period of analysis (about 2 months) the LF was stored at 4 °C. After that, the LF was stored frozen as aliquots of appropriate volume.

The LF was analyzed for soluble carbohydrates by HPAEC with pulse amperometric detection (Dionex ICS 3000 equipped with CarboPac PA1 column). Analysis was performed before and after acid hydrolysis (3% H_2_SO_4_, 1 h at 120 °C), to determine both mono- and oligomeric sugars. Furfural, hydroxymethyl furfural and acetic acid were analyzed by HPLC, using a Bio-Rad Aminex HPX-87H column, with 5-mM H_2_SO_4_ as eluent. Soluble phenolics were determined by UV-absorbance at 215 nm and 280 nm, according to the method for acid-soluble lignin determination described by Goldschmid [64].

Compositional analysis of the solids was performed according to Sluiter et al. [65]. The main components of the solid fraction were glucose (41.5%), xylose (7.3%), lignin (24.5%), and ash (5.8%). The solid fraction was kept frozen in plastic bags.

### Degradation of CNWs by *Sm*LPMO10A

Experiments were made in 50-mM sodium acetate pH 5.0 at 25 °C. Stirring was omitted but the reaction mixture was gently mixed with a pipet before each sampling. The concentration of CNWs was 1.0 g L^−1^ and the concentration of *Sm*LPMO10A was varied between 0.05 and 0.25 µM. The *Sm*LPMO10A was added to the CNWs followed by the addition of LF (pre-incubated at 25 °C for the indicated time) to start the reaction. The amount of added LF corresponded to 5%, 10% or 15% (v/v) of the final reaction volume. At selected time points, 0.1-mL aliquots were withdrawn and mixed with 0.025 mL of 1.0-M NaOH to stop the reaction. Non-labeled CNWs (to 3 g L^−1^) in 0.2-M NaOH were added to improve sedimentation [52] and solids were separated by centrifugation (5 min×10^4^*g*). *Sm*LPMO10A activity was calculated based on the concentration of radioactive soluble products (expressed in *N*-acetylglucosamine equivalents, NAG_eq_) exactly as described in Kuusk et al. [48]. In this previous study, it was established that one *Sm*LPMO10A oxidative cleavage on average leads to release of approximately four NAG_eq_ and this 4 to 1 ratio takes into account that part of the oxidized sites remains in the insoluble substrate [48]. Therefore, the concentration of NAG_eq_ corresponds to the total concentration of monosaccharide equivalents in the soluble fraction and is not dependent on the average degree of polymerization of soluble products (which is known to be around 8 for the *Sm*LPMO10A/CNWs system [48]). In the experiments with HRP, the LF (or solid fraction, see below) was mixed with CNWs. HRP (1.0-µM final concentration) was added to the mixture of CNWs and LF followed by the addition of *Sm*LPMO10A (30 s after the addition of HRP) to start the reaction. In the control experiments without LF or *Sm*LPMO10A, the experiments were made as described above but the LF or *Sm*LPMO10A were replaced with corresponding amount of buffer.

In the experiments for measuring the concentration of H_2_O_2_ in LF upon pre-incubation of LF at 50 °C, the aliquot of pre-incubated LF (to a final concentration of 10% v/v) was added to the mixture of CNWs and *Sm*LPMO10A to start the reaction. The *Sm*LPMO10A reaction was conducted at 25 °C as described above. In some cases, ascorbic acid (0.1-mM final concentration) was added (30 s before the addition of LF) to ensure efficient priming of *Sm*LPMO10A [50]. The concentration of H_2_O_2_ was calculated from the concentration of soluble NAG_eq_ using a previously established stoichiometry of 4 NAG_eq_/H_2_O_2_ [48].

In the experiments with the solid fraction from the hydrothermal pretreatment of wheat straw, 40-mL water (at 4 °C) was added to 5 g of frozen solid fraction. After 30 min, the solids were separated by centrifugation and the pellet was homogenized by grinding in a mortar (at 4 °C) until it was possible to handle the suspension with a pipet. The concentration of solids in homogenized material was measured by weighing (after drying in a rotary evaporator). The experiments with the solid fraction (10 g L^−1^ solids were added to *Sm*LPMO10A reaction) were made exactly as described above for the experiments with LF.

In all cases, the sample for the zero-time point was withdrawn before the addition of LF and was treated as the other samples. The reading of the zero-time point was subtracted from the readings of all time points.

### Degradation of BMCC by *Tr*LPMO9A

Experiments were made in 50-mM sodium acetate pH 5.0 at 25 °C or 50 °C in 1.0-mL total volume. Stirring was omitted but the reaction mixture was gently mixed with a pipet before each sampling. The concentration of BMCC was 0.6 g L^−1^ or 1.2 g L^−1^ and that of *Tr*LPMO9A was varied between 0.1 and 0.5 µM. The LF (pre-incubated at 25 °C or 50 °C for the indicated time) was added to the BMCC and the reaction was started by the addition of *Tr*LPMO9A. The amount of added LF corresponded to 10% or 20% (v/v) of the total reaction volume. At selected time points, 0.2-mL aliquots were withdrawn and the solids were immediately separated by centrifugation (2 min×10^4^*g*). Of note, the stopping by alkali was not suitable because of high and nonstable background readings of the LF BMCC mixtures in the alkaline conditions. The concentration of soluble products (expressed in Glc_eq_) was calculated from the radioactivity readings in the supernatants. For this, the radioactivity readings in the supernatant were first converted into the degree of conversion of BMCC (using total radioactivity of BMCC in the sample) and the degree of conversion of BMCC was converted into the concentration of glucose equivalents (using total glucose in BMCC). In the experiments with HRP, the HRP (1.0-µM final concentration) was added to the mixture of BMCC and LF 1 min before starting the reaction by the addition of *Tr*LPMO9A. In the control experiments without LF or *Tr*LPMO9A, the experiments were made as described above but the LF or *Tr*LPMO9A was replaced with corresponding amounts of buffer. In the experiments where reactions were supplied with H_2_O_2_, the H_2_O_2_ (10–50-µM final concentration) was added to the mixture of BMCC and LF immediately before starting the reaction by adding *Tr*LPMO9A.

In all cases, a sample for the zero-time point was withdrawn just before the addition of *Tr*LPMO9A and was treated as the other samples. The reading of the zero-time point was subtracted from the readings of all time points.

### Pre-incubation of LF before LPMO reaction

The 1.0-mL frozen aliquots of LF in 2.0-mL screw-cap vials were placed in a thermostat bath and incubated at 25 °C or 50 °C for durations ranging from 0.5 to 96 h. Time after time the vials were gently mixed by turning around and the caps were opened (at least once in a day) to allow equilibration with fresh air. Incubation was made in the dark without stirring. The zero time for pre-incubation is the time when the vial with frozen LF was placed (it took few min to melt the material) in the thermostat bath. A small amount of solids in the LF precipitated by gravity and these were not added to the LPMO reactions. After defined pre-incubation times, the LF was added to the LPMO reactions as detailed above.

## Results

### The liquid fraction from hydrothermal pretreatment of wheat straw supports activity of a chitin-active LPMO

**Fig. 1:**
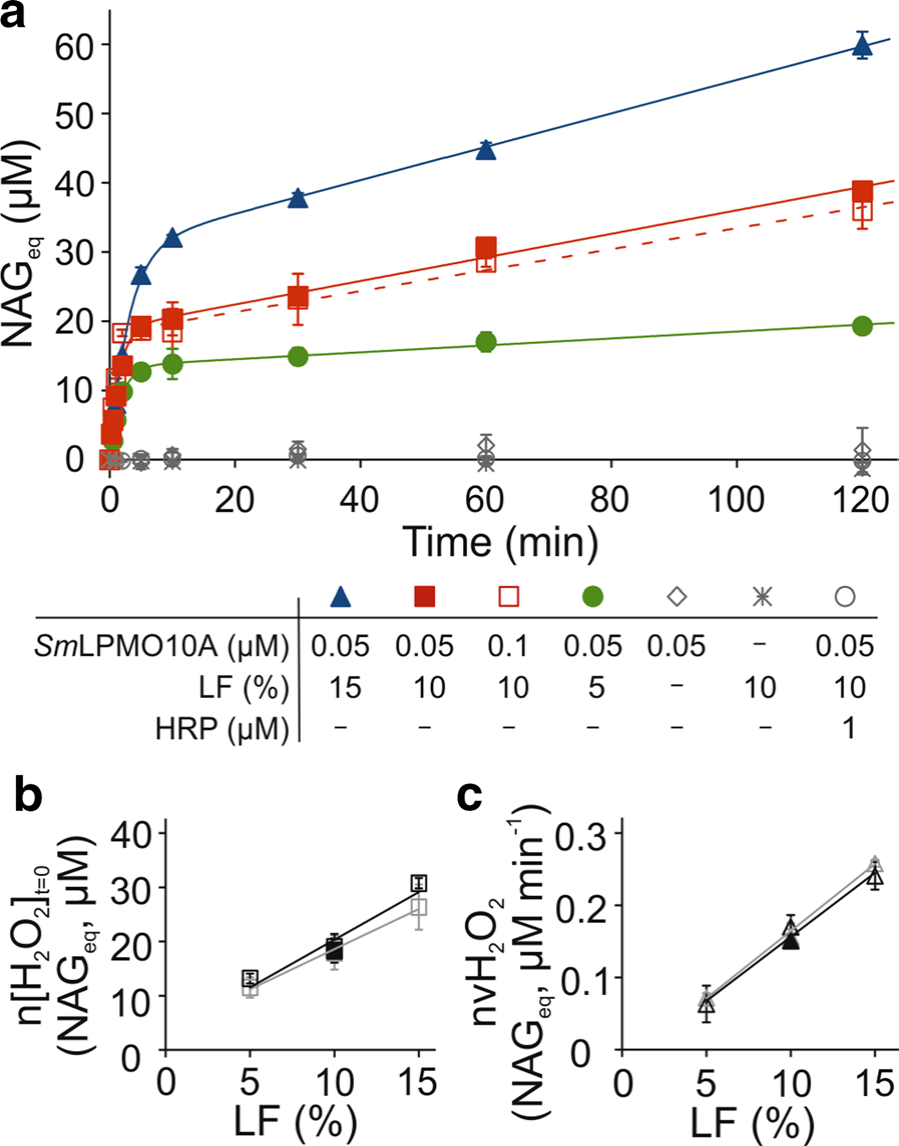
Liquid fraction (LF) supports the degradation of chitin by *Sm*LPMO10A. Reactions were made in 50 mM sodium acetate (pH 5.0) at 25 °C. The concentration of CNWs was 1.0 g L^−1^ and that of *Sm*LPMO10A was 0.05 µM (except in one series where it was 0.1 µM, as indicated in the Figure). Before use in the reactions, the LF was pre-incubated at 25 °C for overnight. The amount of LF was 5%, 10%, or 15% of the total reaction volume. **a** Time curves of the formation of soluble products (expressed in *N*-acetylglucosamine equivalents, NAG_eq_) upon incubation of CNWs with *Sm*LPMO10A in the presence of LF. Solid lines show the best-fit of non-linear regression analysis according to Eq. 1. Control experiments show the results obtained (i) with 10% LF but in the presence of 1-µM horseradish peroxidase (note that no extra HRP substrate was added, since the LF contains HRP substrates), (ii) with 10% LF but in the absence of *Sm*LPMO10A, or (iii) in the absence of LF. Note that no reductant was added to these reaction mixtures. **b**, **c** Dependence of (**b**) *n*[H_2_O_2_]_*t*=0_, and (**c**) $nv_{{{\text{H}}_{2} {\text{O}}_{2} }}$ on the concentration of LF in the LPMO reaction. The values of *n*[H_2_O_2_]_*t*=0_ and $nv_{{{\text{H}}_{2} {\text{O}}_{2} }}$ were found by analysis of the data in **a** using non-linear regression analysis according to Eq. 1 (opened black symbols) or linear regression analysis (only the data points measured between 10 and 120 min were included here) according Eq. 2 (open gray symbols). Filled black symbols represents the data obtained with 10% LF and double the amount (0.1 µM) of *Sm*LPMO10A. Error bars represent S.D. and are from at least two independent measurements

The kinetics of H_2_O_2_-driven degradation of chitin (^14^C-labeled crystalline α-chitin nanowhiskers, CNWs) by *Sm*LPMO10A has been characterized in detail before [48, 50]. Provided with 0.1-mM AscA as reductant, the *k*_cat_ value for oxidation of CNWs was 6.7 oxidative cleavages s^−1^ and the *K*_m_ values for H_2_O_2_ and CNWs are 2.8 µM and 0.58 g L^−1^, respectively. One molecule of H_2_O_2_ supports one oxidative cleavage with concomitant release of 4 soluble *N*-acetylglucosamine equivalents (NAG_eq_) [48]. Different reducing agents like ascorbic acid, gallic acid and methylhydroquinone can support H_2_O_2_-driven oxidation of CNWs by *Sm*LPMO10A [50]. Here, we show that the liquid fraction (LF) from hydrothermal pretreatment of wheat straw (for the composition see Table 1) can also support oxidation of CNWs by *Sm*LPMO10A. Addition of LF to the premixed CNWs and *Sm*LPMO10A resulted in the release of ^14^C-labeled soluble products (expressed in NAG_eq_, Fig. 1a). There was no activity in the control experiments without *Sm*LPMO10A or LF. In line with earlier observations [46, 50], the release of NAG_eq_ were not detected in the presence of horseradish peroxidase (HRP) indicating that the H_2_O_2_ is responsible for the activity of *Sm*LPMO10A under the conditions used. Since no external electron donor (reductant) nor H_2_O_2_ was added, these results suggest that compounds present in LF support *Sm*LPMO10A with both, electrons and the H_2_O_2_ co-substrate.

**Table 1:** Main components of the liquid fraction from hydrothermal pre-treatment of wheat straw

Compound^a^	Concentration (g L^−1^)
Xylose total^b^	4.28
Xylose monomeric	0.37
Acetate	1.4
Hydroxymethyl furfural	< 0.004
Furfural	0.46
UV-phenolics	2.04

^a^LF also contained low amounts of glucose (total 0.53 g L^−1^), arabinose (total 0.34 g L^−1^), fructose (total 0.27 g L^−1^), and galactose (total 0.26 g L^−1^)

^b^The total amount of sugars was measured after acid hydrolysis of LF

Time curves of NAG_eq_ formation were in accordance with the so-called “burst” kinetics, which is characterized by an initial transient burst of activity followed by slow and linear formation of NAG_eq_ in time (Fig. 1a). Under our experimental conditions, the initial burst decayed within the first 10 min; whereas, the linear formation of products in time continued up to the longest time point tested (2 h). One may speculate that the initial activity burst is due to rapid consumption of H_2_O_2_ already present in LF at time zero (i.e., before the addition of LPMO); whereas the linear stage reflects the slow formation of H_2_O_2_ in the reaction mixture (see below). Notably, doubling the concentration of *Sm*LPMO10A (from 0.05 µM to 0.1 µM) had no effect on the rate of NAG_eq_ formation in linear stage. This suggests that H_2_O_2_ is forming in a reaction independent from the LPMO. In the case of low initial H_2_O_2_ concentrations (i.e., conditions where enzyme inactivation can be ignored) [48], Eq. 1 can be used as a first approximation to describe the system.

1 $$\left[ {{\text{NAG}}_{\text{eq}} } \right] = n\left[ {{\text{H}}_{2} {\text{O}}_{2} } \right]_{{\left( {t = 0} \right)}} \left( {1 - {\text{e}}^{{ - k_{{\left( {\text{LPMO}} \right)}}^{\text{obs}} t}} } \right) + nv_{{\left( {{\text{H}}_{2} {\text{O}}_{2} } \right)}} t$$

In Eq. 1, the *n* is an average number of soluble sugars (in monosaccharide equivalents) released per one molecule of H_2_O_2_ consumed by LPMO (for *Sm*LPMO10A with CNWs, *n* = 4), [H_2_O_2_]_(*t*=0)_ is the initial concentration of H_2_O_2_ in the LF, and *t* is time. The $k_{{\left( {\text{LPMO}} \right)}}^{{^{\text{obs}} }}$ is the observed rate constant of LPMO-catalyzed oxidation of polysaccharide. $k_{{\left( {\text{LPMO}} \right)}}^{{^{\text{obs}} }}$ depends not only on the values of kinetic parameters, the concentrations of polysaccharide and LPMO [48] but also on the nature and concentration of the reductant [50]. The $v_{{\left( {{\text{H}}_{2} {\text{O}}_{2} } \right)}}$ is the rate of LPMO-independent formation of H_2_O_2_ in LF. Note that with Eq. 1 we assume that the rate of H_2_O_2_ consumption in polysaccharide oxidation by LPMO is much higher than the rate of its formation/decomposition in LF, so that the $v_{{\left( {{\text{H}}_{2} {\text{O}}_{2} } \right)}}$ is directly reflected in the formation of LPMO products (NAG_eq_). This assumption is plausible since doubling the concentration of *Sm*LPMO10A had no effect on the steady-state rate of the LPMO reaction (Fig. 1a).

The time curves of the release of NAG_eq_ made at different LF concentrations were in accordance with Eq. 1 (Fig. 1a, solid lines). Nonlinear regression analysis was used to find the values of parameters and there was a linear correlation between the concentration of LF and both, *n*[H_2_O_2_]_(*t*=0)_ and $nv_{{\left( {{\text{H}}_{2} {\text{O}}_{2} } \right)}}$ (Fig. 1b, c). On the other hand both, *n*[H_2_O_2_]_(*t*=0)_ and $nv_{{\left( {{\text{H}}_{2} {\text{O}}_{2} } \right)}}$ were independent of the concentration of *Sm*LPMO10A. These results are in accordance with the model whereby the kinetics of LF-driven degradation of CNWs by *Sm*LPMO10A is governed by the H_2_O_2_ initially present in LF and that formed in LF, in an LF-dependent but *Sm*LPMO10A-independent manner.

### H_2_O_2_ is formed in LF but does not accumulate to high levels upon incubation of LF in aerobic conditions

Sensitive detection of H_2_O_2_ above a background in a redox-active environment such as LF, with a myriad of compounds and ongoing reactions, is a challenging task. Therefore, we exploited the dependency of the kinetics of the *Sm*LPMO10A/CNW system on available H_2_O_2_ and used the kinetics of LPMO catalysis with a ^14^C-labeled polymeric substrate for sensing, not only the concentration but also the rate of the formation of H_2_O_2_ in the LF. First, we were interested in if and how [H_2_O_2_]_(*t*=0)_ and $v_{{\left( {{\text{H}}_{2} {\text{O}}_{2} } \right)}}$ change upon incubation of LF in aerobic conditions. For that, we pre-incubated LF at 25 °C in the dark without stirring for time-periods ranging from 0.5 to 96 h. Zero time for pre-incubation is the time when the frozen vial with LF was placed at 25 °C. After pre-incubation, an aliquot of LF was added (10% v/v) to the mixture of CNWs and *Sm*LPMO10A, and the formation of NAG_eq_ in time was followed, which was then used to calculate [H_2_O_2_]_(*t*=0)_ and $v_{{\left( {{\text{H}}_{2} {\text{O}}_{2} } \right)}}$. Here, only the linear regions of NAG_eq_ formation in time were assessed (from 10 min to 2 h). The $k_{{\left( {\text{LPMO}} \right)}}^{{^{\text{obs}} }}$ values found from the analysis of the data in Fig. 1a were in the order of 0.6 min^−1^. This translates to the half-life of [H_2_O_2_]_(*t*=0)_ in the LPMO reaction of around 1 min. After 10 min of *Sm*LPMO10A reaction, the exponential term is close to zero (as visible in Fig. 1a) and Eq. 1 simplifies to:

2 $$\left[ {{\text{NAG}}_{\text{eq}} } \right] = n\left[ {{\text{H}}_{2} {\text{O}}_{2} } \right]_{{\left( {t = 0} \right)}} + nv_{{\left( {{\text{H}}_{2} {\text{O}}_{2} } \right)}} t$$

Provided that *n* and $v_{{\left( {{\text{H}}_{2} {\text{O}}_{2} } \right)}}$ is time invariant, Eq. 2 can be analyzed using linear regression. This simplified approach is justified, as the *n*[H_2_O_2_]_(*t*=0)_ and $nv_{{\left( {{\text{H}}_{2} {\text{O}}_{2} } \right)}}$ values found using full progress curves and analysis according to Eq. 1 were very similar to the values found when using Eq. 2 (Fig. 1b, c).

**Fig. 2:**
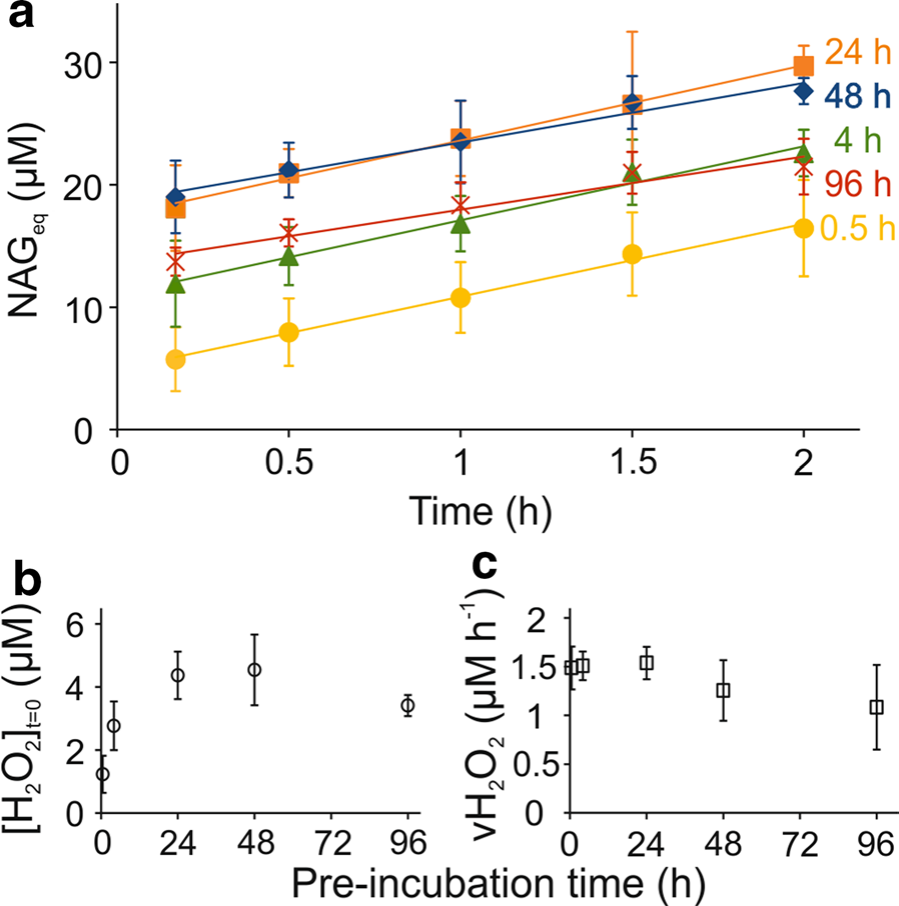
Dependence of the [H_2_O_2_]_*t*=0_ and $v_{{\left( {{\text{H}}_{2} {\text{O}}_{2} } \right)}}$ on pre-incubation time of LF at 25 °C. Reactions were made in 50 mM sodium acetate (pH 5.0) at 25 °C. The concentration of CNWs was 1.0 g L^−1^ and that of LF was 10% (v/v). **a** Time curves of the formation of soluble products (expressed in *N*-acetylglucosamine equivalents, NAG_eq_) upon incubation of CNWs with *Sm*LPMO10A in the presence of LF that was pre-incubated at 25 °C for different times (as indicated in the plot). The graphs shows average values, and S.D, obtained from experiments with *Sm*LPMO10A concentrations of 0.05 µM, 0.1 µM, or 0.25 µM (see Additional file 1: Fig. S2 for the individual curves). Solid lines show linear regression of the data according to Eq. 2. **b**, **c** Dependence of (**b**) the [H_2_O_2_]_*t*=0_ and (**c**) the $v_{{\left( {{\text{H}}_{2} {\text{O}}_{2} } \right)}}$, on pre-incubation time of LF. The values of [H_2_O_2_]_*t*=0_ and $v_{{\left( {{\text{H}}_{2} {\text{O}}_{2} } \right)}}$ were calculated from the parameter values of linear regression analysis of the data in **a** according to Eq. 2 using the stoichiometry (*n*) of 4 NAG_eq_/H_2_O_2_ for the *Sm*LPMO10A/CNWs system [48]. Error bars represent S.D. and are from three independent measurements each made in single parallel but at different concentration of *Sm*LPMO10A (Additional file 1: Fig. S2)

Figure 2a shows time curves for NAG_eq_ formation over time in reactions with LF subjected to varying periods of pre-incubation. For each time of LF pre-incubation, the time curves of NAG_eq_ formation were measured using three different (each in single parallel) *Sm*LPMO10A concentrations (0.05, 0.1, and 0.25 µM). Since no clear dependence on LPMO concentration was found (Additional file 1: Fig. S2), average results obtained with different enzyme concentrations are shown in Fig. 2a. The increase in [NAG_eq_] was linear in time regardless of the pre-incubation time of LF. However, the intercept increased, while the slope slightly decreased with increasing pre-incubation time. Next we converted the values of intercepts to the values of [H_2_O_2_]_(*t*=0)_ and the values of slopes to the values of $v_{{\left( {{\text{H}}_{2} {\text{O}}_{2} } \right)}}$ using the known stoichiometry of *Sm*LPMO10A reaction (*n* = 4; so 4 NAG_eq_ are produced per H_2_O_2_). The dependence of [H_2_O_2_]_(*t*=0)_ and $v_{{\left( {{\text{H}}_{2} {\text{O}}_{2} } \right)}}$ on pre-incubation time is shown in Fig. 2b and c, respectively. The rate of H_2_O_2_ formation in LF slightly decreased with the pre-incubation time of LF and was in the order of 1.0–1.5 µM h^−1^ (Fig. 2c). The concentration of H_2_O_2_ in LF increased upon pre-incubation of LF but seemed to level off around 3–5 µM in longer pre-incubations (Fig. 2b). Importantly, the levels of H_2_O_2_ in LF were much lower than those expected based on the rate of its formation. As an example, with a rate around 10 µM h^−1^ (note that we here extrapolate the rate of H_2_O_2_ formation measured in 10% LF to what is expected in case of 100% LF, assuming a linear correlation between $v_{{\left( {{\text{H}}_{2} {\text{O}}_{2} } \right)}}$ and [LF]), about 1.0-mM H_2_O_2_ would be formed upon pre-incubation of LF for 96 h. However, the measured [H_2_O_2_]_(*t*=0)_ after 96-h pre-incubation was just 34 ± 3 µM (measured using 10% LF, but extrapolated to 100% LF assuming a linear correlation between $[{\text{H}}_{2} {\text{O}}_{2} ]_{{\left( {{\text{H}}_{2} {\text{O}}_{2} } \right)}}$ and [LF]). These results suggest that H_2_O_2_ is an intermediate of LF oxidation.

Of note, the solid fraction from hydrothermal pre-treatment of wheat straw also supported *Sm*LPMO10A activity. An experiment with 10 g L^−1^ solid fraction at 25 °C, pH 5.0 (Additional file 1: Fig. S3) yielded an estimated H_2_O_2_ production rate of 1.4 µM h^−1^.

### LF from hydrothermal pretreatment of wheat straw supports activity of a cellulose-active LPMO

**Fig. 3:**
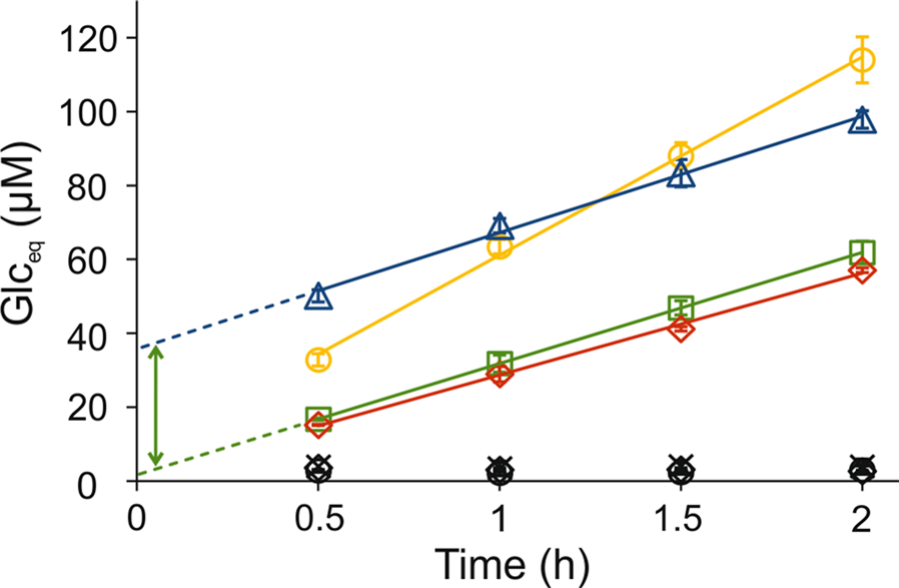
Liquid fraction (LF) supports the degradation of cellulose (BMCC) by *Tr*LPMO9A. Reactions were made in 50 mM sodium acetate (pH 5.0) at 50 °C. The concentration of BMCC was 0.6 g L^−1^ (except in one series where it was 1.2 g L^−1^, red diamonds). Before use in the LPMO reaction, the LF was pre-incubated at 50 °C for 0.5 h. All colored points and lines refer to reactions with LF with a concentration 10%, except for the yellow series where it was 20% (v/v). In summary: green squares, base case; red diamonds, double BMCC concentration; yellow circles, double LF. The blue triangles are from an experiment where the concentration of LF was 10% but the reactions were supplied with 20-µM H_2_O_2_ at *t* = 0. The green arrow shows the increase in intercept upon supplementation of the reaction with 20-µM H_2_O_2_. The graphs shows the average values, and S.D, obtained from experiments with *Tr*LPMO9A concentrations (each in single parallel) of 0.1 µM, 0.2 µM, or 0.5 µM (see Additional file 1: Fig. S5 for the individual curves). The data points show the formation of soluble products (expressed in glucose equivalents, Glc_eq_). Solid lines show linear regression of the data according to Eq. 2. Control experiments show the results obtained (i) with 10% LF but in the presence of 1 µM HRP (black circles), (ii) with 10% LF but in the absence of *Tr*LPMO9A (black diamonds), or (iii) in the absence of LF (black crosses)

Since LPMOs are important components of commercial cellulolytic cocktails, we were interested in whether the LF can support LPMOs also at 50 °C, a more relevant temperature for industrial applications. Unfortunately, the use of 50 °C was not compatible with *Sm*LPMO10A. Therefore, we used a cellulose-active LPMO of *Trichoderma reesei* (*Tr*LPMO9A, formerly *Tr*Cel61A) [17, 66–69] and ^14^C-labeled bacterial microcrystalline cellulose (BMCC), to measure the [H_2_O_2_]_(*t*=0)_ and $v_{{\left( {{\text{H}}_{2} {\text{O}}_{2} } \right)}}$ at 50 °C. The LF indeed supported *Tr*LPMO9A in releasing soluble products, the concentration of which was expressed in glucose equivalents (Glc_eq_), from BMCC at 50 °C (Fig. 3). In all cases, the increase in [Glc_eq_] was linear over time during the measurement period (from 0.5 to 2 h) (Fig. 3) and the results were analyzed using Eq. 2. Most importantly, the slopes ($nv_{{\left( {{\text{H}}_{2} {\text{O}}_{2} } \right)}}$) and intercepts (*n*[H_2_O_2_]_(*t*=0)_) were independent on the concentrations of *Tr*LPMO9A and BMCC. On the other hand, both slope and intercept increased with increasing concentration of LF. Supplementation of the reactions with HRP (1.0 µM) totally abolished the release of soluble products and no radioactivity was released in experiments without *Tr*LPMO9A or LF (Fig. 3). Supplementation of the *Tr*LPMO9A/BMCC/LF reactions with H_2_O_2_ (20 µM) caused an activity burst that was reflected in an increased intercept but had no effect on the rate of further Glc_eq_ formation (0.50 ± 0.03 versus 0.52 ± 0.05 µM Glc_eq_ min^−1^) (Fig. 3). Collectively, these results suggest that the formation of H_2_O_2_ governs the steady-state rate of soluble product formation without being dependent on *Tr*LPMO9A or cellulose concentration, while the initial activity burst is caused by the H_2_O_2_ present in the LF before the addition of LPMO.

### Stoichiometry of *Tr*LPMO9A reaction

**Fig. 4:**
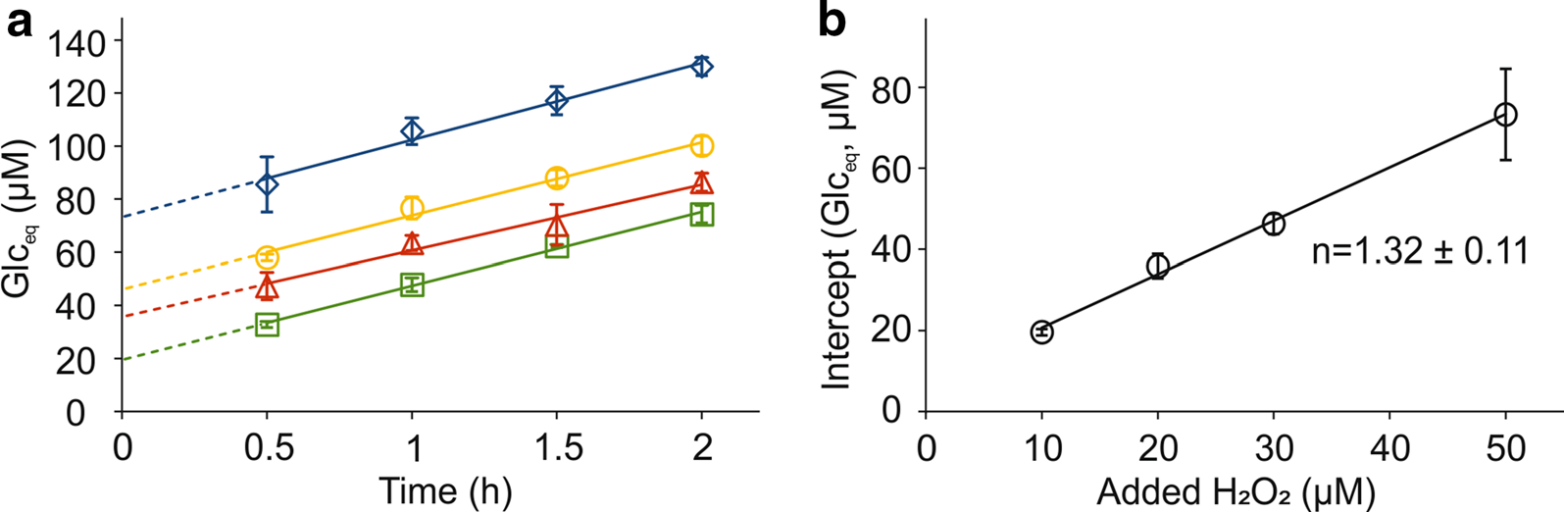
Stoichiometry of the *Tr*LPMO9A reaction at 50 °C. Reactions were made in 50 mM sodium acetate (pH 5.0) at 50 °C. The concentrations of BMCC, LF and *Tr*LPMO9A were 0.6 g L^−1^, 10% (v/v) and 0.5 µM, respectively. Before use in the LPMO reaction, the LF was pre-incubated at 50 °C for 24 h. All reactions were supplemented with H_2_O_2_ (10–50 µM, final concentrations) just before starting the reaction by addition of *Tr*LPMO9A. Error bars represent S.D. and are from at least two independent measurements. **a** Time curves for the formation of soluble products (expressed in glucose equivalents, Glc_eq_) upon incubation of BMCC with *Tr*LPMO9A in the presence of externally added H_2_O_2_. Solid lines show linear regression of the data according to Eq. 2. **b** Dependency of intercept values obtained from linear regression of the data shown in panel A on the concentration of added H_2_O_2_. The solid line shows linear regression of the data and the slope of the line suggests stoichiometry of 1.32 ± 0.11 Glc_eq_/H_2_O_2_ for the *Tr*LPMO9A/BMCC system

To derive the values of [H_2_O_2_]_(*t*=0)_ and $v_{{\left( {{\text{H}}_{2} {\text{O}}_{2} } \right)}}$ from the kinetics of Glc_eq_ formation, one must know an average number of Glc_eq_ released per one molecule of H_2_O_2_ consumed (i.e., parameter *n* in Eq. 2). The *n* can be measured through detailed kinetic characterization of H_2_O_2_-driven degradation of polysaccharides as has been done for *Sm*LPMO10A [48]. Unfortunately, the specific radioactivity of our BMCC preparation was not sufficiently high to permit detailed kinetic characterization of its H_2_O_2_-driven degradation by *Tr*LPMO9A. Therefore, we estimated the value of *n* using alternative approaches. Comparison of the rates of NAG_eq_ formation measured using the *Sm*LPMO10A/CNWs system and Glc_eq_ formation measured using the *Tr*LPMO9A/BMCC system suggested *n* = 2.1 ± 0.3 for the *Tr*LPMO9A/BMCC system (Additional file 1: Fig. S4) at 25 °C. An increase in intercept upon supplementation of *Tr*LPMO9A/BMCC/LF reactions with H_2_O_2_ (Fig. 3) provides an alternative approach for measuring stoichiometry. Of note, the latter approach can also be used at 50 °C. Supplementation of *Tr*LPMO9A/BMCC/LF reactions (before the experiment the LF was pre-incubated at 50 °C for 24 h) with H_2_O_2_ (10–50 µM) caused an increase in the initial burst of Glc_eq_ release with no influence on the later, linear release of Glc_eq_ in time (Fig. 4a). Intercept values obtained from linear regression analysis of data in Fig. 4a scaled linearly with the concentration of added H_2_O_2_ (Fig. 4b). The slope of this linear dependency suggested the value of *n* = 1.32 ± 0.11 for the *Tr*LPMO9A/BMCC system at 50 °C and this figure was used throughout this study. Note, that the *n* shall not be confused with the average degree of polymerization of soluble products since the latter depends on the probability of an oxidized group being in soluble fraction, which is 0.5 for *Sm*LPMO10A/CNW [48] but not known for the *Tr*LPMO9A/BMCC system. Regarding the purposes of this study, it is important and enough to know that an average of 1.32 soluble Glc_eq_ are released from BMCC per one molecule of H_2_O_2_ consumed by *Tr*LPMO9A.

### Rate of H_2_O_2_ formation and accumulation upon incubation of LF at 50 °C

**Fig. 5:**
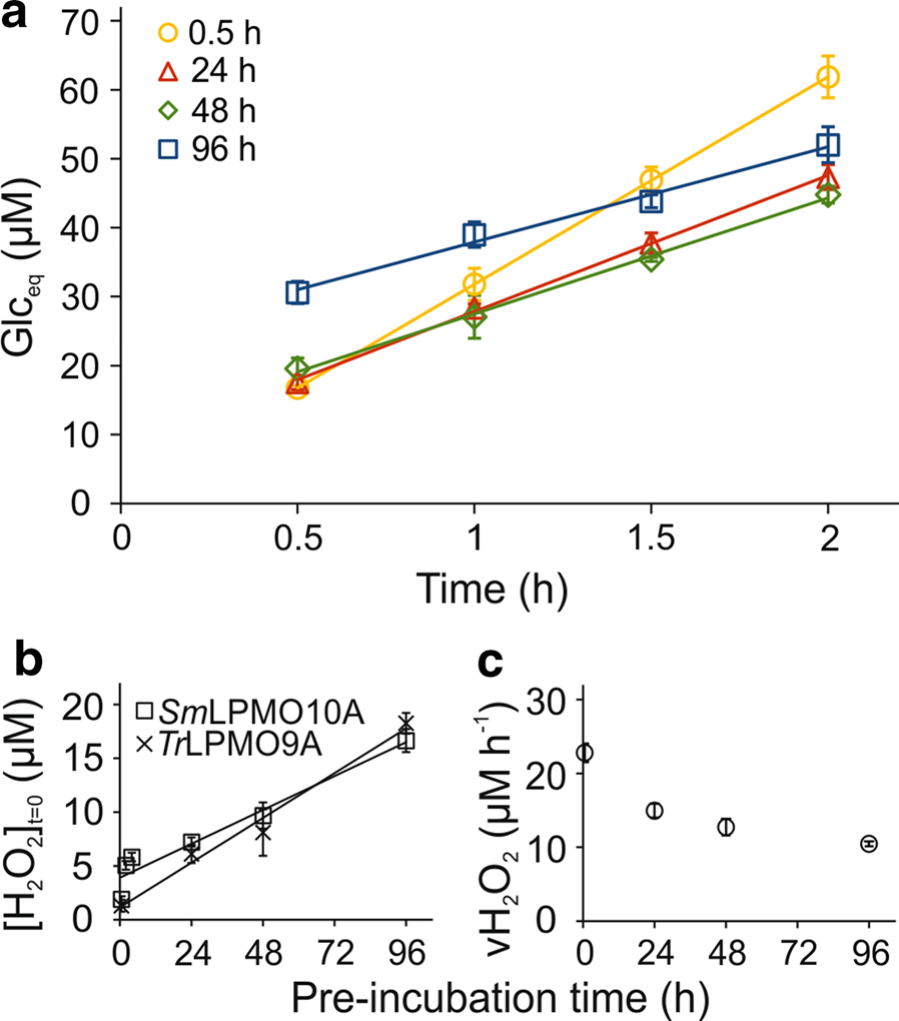
Dependence of the [H_2_O_2_]_*t*=0_ and $v_{{\left( {{\text{H}}_{2} {\text{O}}_{2} } \right)}}$ on pre-incubation time of LF at 50 °C. Reactions were made in 50 mM sodium acetate (pH 5.0) at 50 °C. The concentration of BMCC was 0.6 g L^−1^ and that of LF was 10% (v/v). **a** Time curves of the formation of soluble products (expressed in glucose equivalents, Glc_eq_) upon incubation of BMCC with *Tr*LPMO9A in the presence of LF that was pre-incubated at 50 °C for different time (as defined on the plot). Shown are average values, and S.D, obtained from experiments with *Tr*LPMO9A concentrations of 0.1 µM, 0.2 µM, or 0.5 µM (see Additional file 1: Fig. S5 for the individual curves). Solid lines show linear regression of the data according to Eq. 2. **b**, **c** Dependence of (**b**) the [H_2_O_2_]_*t*=0_ and (**c**) the $v_{{\left( {{\text{H}}_{2} {\text{O}}_{2} } \right)}}$, on pre-incubation time of LF at 50 °C. The values of [H_2_O_2_]_*t*=0_ and $v_{{\left( {{\text{H}}_{2} {\text{O}}_{2} } \right)}}$ were found by fitting the data in **a** to Eq. 2 and using a stoichiometry of 1.32 Glc_eq_/H_2_O_2_. The values of [H_2_O_2_]_*t*=0_ were also measured using an alternative approach (designated with *Sm*LPMO10A on **b**) where LF was pre-incubated at 50 °C but LPMO reaction was done at 25 °C using the *Sm*LPMO10/CNW system and short reaction times (Additional file 1: Fig S7). Solid lines in panel B show best-fits of the linear regression analysis. Error bars represent S.D. and are from three independent measurements each made at different concentration of *Tr*LPMO9A (Additional file 1: Fig. S5)

To assess redox properties under typical industrial bioprocessing conditions, we pre-incubated LF at 50 °C in the dark without stirring for time-periods ranging from 0.5 to 96 h, aerobically. After pre-incubation, an aliquot of LF was added (10% or 20% v/v) to BMCC followed by the addition of *Tr*LPMO9A to start the LPMO reaction. The formation of Glc_eq_ was linear in time (Fig. 5a) and the rate of Glc_eq_ formation was independent of the concentration of *Tr*LPMO9A (Additional file 1: Fig. S5). The time curves were fitted to Eq. 2 and the values of slopes and intercepts were converted to the values of $v_{{\left( {{\text{H}}_{2} {\text{O}}_{2} } \right)}}$ and [H_2_O_2_]_(*t*=0),_ respectively, using *n* = 1.32. The [H_2_O_2_]_(*t*=0)_ increased (Fig. 5b) while $v_{{\left( {{\text{H}}_{2} {\text{O}}_{2} } \right)}}$ decreased (Fig. 5c) with pre-incubation time of LF. Corresponding results obtained with 20% LF in the LPMO reaction are shown in Additional file 1: Fig. S6. Note that the rate of H_2_O_2_ formation in LF is strongly dependent on temperature. The $v_{{\left( {{\text{H}}_{2} {\text{O}}_{2} } \right)}}$ values derived from experiments with 0.5-h pre-incubation and 10% LF were 1.5 ± 0.2 µM h^−1^ (Fig. 2c) and 22.7 ± 1.2 µM h^−1^ (Fig. 5c) at 25 °C and 50 °C, respectively. This difference in rates translates to a Q_10_ value of 3.0.

We note that determination of [H_2_O_2_]_(*t*=0)_ values using the values of intercepts obtained by analysis of linear regions of progress curves according to Eq. 2 may be complicated for the systems with low [H_2_O_2_]_(*t*=0)_ and high $v_{{\left( {{\text{H}}_{2} {\text{O}}_{2} } \right)}}$ values. Obviously, the high concentration of H_2_O_2_ produced during LPMO reaction affects precise measurement of low intercept values (see data with 0.5-h pre-incubation of LF in Fig. 5a as an example). Therefore, the [H_2_O_2_]_(*t*=0)_ values were also measured using an alternative approach where the LF was pre-incubated at 50 °C but the LPMO reaction was made at 25 °C using the *Sm*LPMO10A/CNW system and very short reaction times (up to 10 min). In these conditions, the amount of H_2_O_2_ produced during LPMO reaction is negligible compared to its initial amount and Eq. 1 simplifies to Eq. 3.

3 $$\left[ {{\text{NAG}}_{\text{eq}} } \right] = n\left[ {{\text{H}}_{2} {\text{O}}_{2} } \right]_{{\left( {t = 0} \right)}} \left( {1 - {\text{e}}^{{ - k_{{\left( {\text{LPMO}} \right)}}^{\text{obs}} t}} } \right)$$

The time curves of NAG_eq_ formation were analyzed using non-linear regression according to Eq. 3 (Additional file 1: Fig. S7) and the [H_2_O_2_]_(*t*=0)_ values were found from the plateau values of NAG_eq_ formation using *n* = 4. Of note, the shape of the time curves suggested that the *Sm*LPMO10A priming reduction efficiency of LF decreased with pre-incubation of LF and 100-µM ascorbic acid was added to ensure efficient priming (Additional file 1: Fig. S7). The [H_2_O_2_]_(*t*=0)_ found using short times of LPMO reaction and analysis according to Eq. 3 were similar to those found using longer LPMO reactions and analysis according to Eq. 2 (Fig. 5b). In both cases, the [H_2_O_2_]_(*t*=0)_ seemed to scale linearly with pre-incubation time with the slope (i.e. the rate of H_2_O_2_ accumulation in LF during pre-incubation) about 0.15-µM H_2_O_2_ h^−1^ (or 1.5-µM H_2_O_2_ h^−1^ when extrapolated to 100% LF) (Fig. 5b). This rate of H_2_O_2_ accumulation in LF is far lower than the rate of its formation in LF (Fig. 5c), suggesting that H_2_O_2_ is an intermediate in LF oxidation, supporting the conclusion derived from the pre-incubation experiments at 25 °C with the *Sm*LPMO10A/CNW system (Fig. 2).

## Discussion

Since their discovery as oxidative enzymes [5], LPMOs have been a subject of intensive research. Still, the nature of the true co-substrate of LPMOs is a matter of scientific debate. The nature of the co-substrate (i.e., O_2_ or H_2_O_2_) that governs the LPMO kinetics under our experiment conditions is of utmost importance regarding the interpretation of the data presented here. Most importantly, we observed that LF-driven formation of soluble products from cellulose was independent of the concentration of *Tr*LPMO9A (Fig. 3 and Additional file 1: Fig. S5) and the concentrations of cellulose substrate (Fig. 3) in the concentration range studied. This suggests that the rate-limiting step for the release of soluble products from cellulose is independent of the LPMO, a suggestion which is supported by the observation that the rate of chitin degradation by *Sm*LPMO10A also was independent of the enzyme concentration.

A number of different scenarios have been proposed for O_2_-driven degradation of polysaccharides by LPMOs [27, 28] but in all these cases, the rate is expected to depend on enzyme and/or polysaccharide concentration. Catalysis involving insoluble polysaccharides takes place at a solid–liquid interface and two saturation scenarios are possible, saturation of the enzyme with substrate (as in the conventional Michaelis–Menten mechanism) and saturation of substrate with enzyme (also known as the inversed Michaelis–Menten mechanism) [70]. Further increase of substrate concentration in the conditions where enzyme is already saturated with substrate will not increase the rate; however, increasing the enzyme concentration in these conditions should increase the rate. On the other hand, a further increase of the enzyme concentration in conditions where binding sites on the polymer surface are saturated with enzyme will not increase the rate; however, increase of the substrate concentration under such conditions should increase the rate. Thus, our observations cannot be ascribed to saturating conditions.

**Fig. 6:**
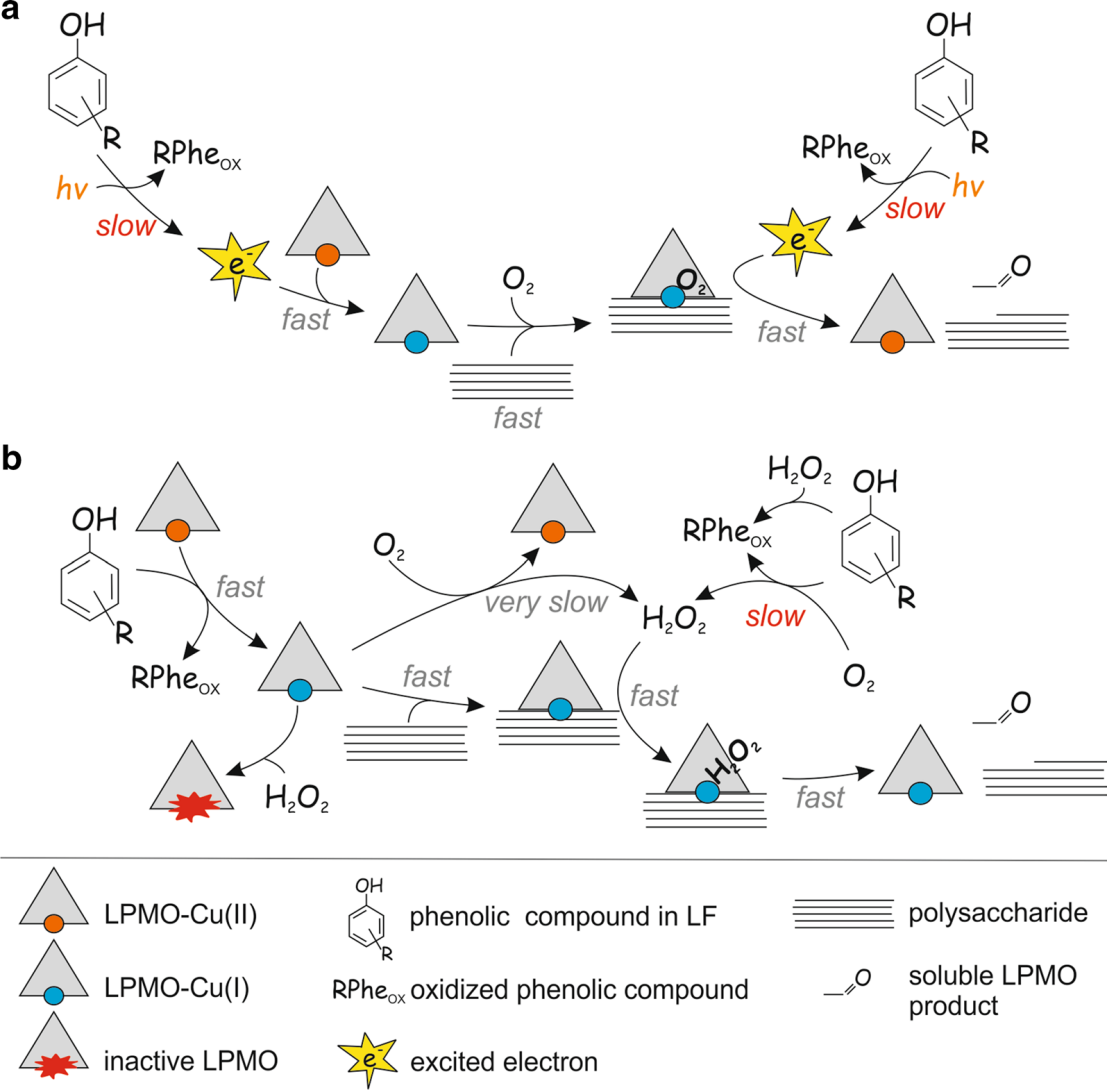
Possible mechanisms of liquid fraction (LF)-driven degradation of polysaccharides by LPMO. **a** According to this mechanism, LPMO uses O_2_ co-substrate and the LF (represented by a phenolic compound) drives the LPMO reaction by generating excited electrons that are used in a monooxygenase reaction. An analogous mechanism has been proposed by Cannela et al. to explain the LPMO activity-boosting effect of light in the presence of pigments [71]. The excited electrons generated in the LF are used for the initial reduction of the LPMO and subsequent catalysis via an LPMO/polysaccharide/O_2_ ternary complex and including delivery of a second electron. The generation of excited electrons in LF may be stimulated (e.g., by light as shown in the scheme by *hν*) but stimulation is not a necessary assumption here. Note that many different mechanisms have been proposed for O_2_-driven catalysis but all these mechanisms assume the delivery of two electrons per one cleavage of glycosidic bond [27, 28]. **b** According to this mechanism the LPMO uses H_2_O_2_ as co-substrate and the LF drives LPMO reaction by generating H_2_O_2_. The slow, LPMO-independent, formation of H_2_O_2_ in the reaction between O_2_ and LF is rate-limiting for LPMO catalysis. This mechanism also assumes the delivery of electrons by LF but here the electrons are used only in the “priming reduction” of the LPMO (from Cu(II) to Cu(I) form). Primed LPMO can catalyze a number of oxidative cleavages of glycosidic bonds until the polysaccharide free LPMO happens to be re-oxidized, either by O_2_ or H_2_O_2_ [46, 48, 50]. Note that re-oxidation of the LPMO by H_2_O_2_ may lead to irreversible inactivation. Re-oxidation of a reduced LPMO by O_2_ may also generate H_2_O_2_ [73]. However, in our experiments, the rates of the routes involving LPMO re-oxidation must have been insignificant compared to the rate of formation of soluble LPMO products since product formation was independent of the LPMO concentration

Cannella et al. proposed that pigment-derived excited electrons are responsible for the boosting effect of light on the degradation of cellulose by LPMOs [71, 72] Therefore, one may speculate that formation of “excited electrons” in LF is responsible for supporting LPMO activity as depicted in Fig. 6a. This scenario would be in accordance with the observed independency of the reaction rate on the concentration of LPMO and polysaccharide as the formation of such “excited electrons” could be a rate-limiting intrinsic property of the LF (note that reaction rates do depend on the amount of LF, Figs. 1, 3). Inhibition of LPMO by HRP can, in principle, be explained by the use of these electrons in the HRP reaction. However, such an “excited electron” scenario cannot explain the activity burst observed upon supplementation of an LPMO reaction with H_2_O_2_ (Figs. 3, 4). Importantly, the observed increase in product formation upon addition of H_2_O_2_ scaled linearly with the concentration of added H_2_O_2_ (Fig. 4b). Further considering the results of the experiment with added H_2_O_2_, it is difficult to explain how a strong oxidant like H_2_O_2_ can support the formation of strong reductants like “excited electrons”.

On the other hand, our observations can readily be explained in the light of H_2_O_2_-driven LPMO catalysis (Fig. 6b). According to this scenario, the release of soluble LPMO products is governed by the H_2_O_2_ present in LF before the addition of the LPMO (burst, c.f [H_2_O_2_]_(*t*=0)_) and by H_2_O_2_ formed during the LPMO reaction (steady-state, c.f $v_{{\left( {{\text{H}}_{2} {\text{O}}_{2} } \right)}}$). It is well known that polysaccharide-free LPMOs [46, 73], including *Tr*LPMO9A [67, 74], can produce H_2_O_2_ in a futile oxidase reaction with O_2_. Importantly, the contribution of this route must be insignificant under our experimental conditions, as in this case the rate is expected to increase with increasing concentration of LPMO. All in all, our results suggest that the LPMO kinetics measured here reflect the presence of H_2_O_2_ and the rate of H_2_O_2_ formation in LF.

Numerous reports have shown that process samples of lignocellulose refining support LPMO activity [7, 30, 31, 36–38]. The positive effect on LPMO activity has been assigned to the electron donating ability of lignin and lignin-derived, mostly phenolic compounds. Here, we show that the LPMO supporting activity of LF is related not only to electron transfer to the LPMO but also to production of the LPMO co-substrate, H_2_O_2_. The large discrepancy between the rate of H_2_O_2_ formation (Figs. 2c, 5c) and H_2_O_2_ accumulation in LF (Figs. 2b, 5b) shows that H_2_O_2_ is an intermediate in LF oxidation. It is plausible that phenolic compounds present in LF are responsible for H_2_O_2_ scavenging [75]. This “H_2_O_2_ buffering” capacity is important regarding the stability of LPMOs as it prevents the accumulation of H_2_O_2_ at high concentrations that may lead to inactivation of the LPMO [46, 48, 76]. Still, the “first contact” of LPMOs with pre-treated biomass slurry may result in inactivation of a significant amount of LPMO. The concentration of H_2_O_2_ in LF (pre-incubated at 50 °C for 24 h) was about 70 µM (Fig. 5b, extrapolated to 100% LF). For example, H_2_O_2_-driven inactivation of polysaccharide-free *Sm*LPMO10A proceeds with a second-order rate constant in the order of 10^3^ M^−1^ s^−1^ (at 25 °C, pH 6.1) [48]. This translates to a half-life of only about 10 s for polysaccharide free *Sm*LPMO10A in 70-µM H_2_O_2_. Therefore, a significant fraction of the LPMO may be inactivated at the very start of the reaction, before binding of the LPMO to the polysaccharide substrate and LPMO-catalyzed reduction of [H_2_O_2_]. Obviously, more kinetic data about H_2_O_2_-driven catalysis and inactivation of LPMOs along with knowledge of H_2_O_2_ concentrations in different biomass slurries are needed, to fully unravel this complex interplay of factors. It is worth noting that in all experiments in this study, LPMO activity was independent of the LPMO concentration, meaning that, even if some LPMO inactivation occurred, there was enough active enzyme left to utilize the available H_2_O_2_.

The rate of H_2_O_2_ production in LF decreased with pre-incubation time of LF under aerobic conditions but did not approach zero within the time frame of the experiment (Fig. 5c and Additional file 1: Fig. S6B). One may speculate that some compounds responsible for H_2_O_2_ production were depleted during the pre-incubation of LF at 50 °C, whereas the concentration of other compounds remained near-constant. A detailed analysis of the very initial phase of the LF-driven LPMO reaction (Additional file 1: Fig. S7) showed that the priming reduction of *Sm*LPMO10A becomes less efficient upon pre-incubation (c.f oxidation) of LF at 50 °C. This indicates that the compounds in LF that are depleted during the pre-incubation may also be good priming reductants of LPMO. However, the precise chemical nature of the compounds in LF that are responsible for the LPMO priming reduction and H_2_O_2_ production/depletion remains to be studied. Although we removed divalent metals from substrates and buffers using treatment with EDTA and Chelex^®^ 100 resin, metal ions possibly present in LF [77] may contribute to the rate of H_2_O_2_ formation and H_2_O_2_ stability in LF.

Of note, the liquid fractions of hydrothermal pretreatment can be inhibitory for glycoside hydrolases. The main components responsible for the inhibition are hemicelluloses-derived oligosaccharides [4, 42, 78, 79], but the inhibition by phenolic compounds has also been demonstrated [4, 42, 43, 45, 79, 80]. Therefore, the overall effect of liquid fraction on lignocellulose degradation depends on the relative contributions of its inhibiting and activating effects, which in turn depend on the composition of the enzyme cocktail and the type of pretreatment. The closest alternative to hydrothermal pretreatment is dilute acid treatment, and generally similar effects could be expected from the corresponding liquid fractions. Pretreatments that lead to extensive delignification may not support LPMO activity since the soluble lignin-containing liquor is usually removed before enzymatic hydrolysis. Based on the present findings, further studies of the relationships between the method of pretreatment and LPMO activity in subsequent enzymatic processing are of interest.

The rate of H_2_O_2_ formation in LF was strongly dependent on temperature with an estimated Q_10_ around 3.0. The Q_10_ value found here seems to be in accordance with a recently reported effect of temperature on the half-life of O_2_ in a slurry of hydrothermally pre-treated wheat straw [81]. From the data in Peciulyte et al., one can estimate a half-life of O_2_ of about 1 h at 50 °C [81]. This translates to a rate constant of O_2_ consumption of 0.69 h^−1^, which in turn translates to a rate of O_2_ consumption of 140 µM h^−1^ (assuming an ambient O_2_ concentration of 0.2 mM). This value is in the same range as the rates of H_2_O_2_ formation in LF at 50 °C shown in Fig. 5c (after extrapolation to 100% LF). Thus, the data on H_2_O_2_ formation in LF from the hydrothermal pre-treatment of wheat straw are in general accordance with the data of O_2_ consumption by the whole slurry of hydrothermally pre-treated wheat straw. Interestingly, Peciulyte et al. showed that the abiotic consumption of O_2_ in slurry was much faster than the diffusion of O_2_ into the slurry, suggesting that the availability of O_2_ may be rate-limiting for the oxidation of biomass components [81]. Apparently, the availability of O_2_ along with the amounts and chemical nature of the components in biomass slurries are key determinants of the rate of H_2_O_2_ formation and, thus, of LPMO activity and stability in biomass processing.

## Conclusions

In this study, we have demonstrated that soluble compounds in the liquid fraction (LF) from hydrothermal pre-treatment of wheat straw support LPMOs with both, electrons and the H_2_O_2_ co-substrate under conditions that are commonly used in enzymatic biomass processing. Both, a bacterial chitin-active and a fungal cellulose-active LPMO were supported by LF and the H_2_O_2_ was produced in the LF in an LPMO-independent manner. Our results point to that H_2_O_2_ is an intermediate and not an end product in LF oxidation. This is important, since the further reduction of H_2_O_2_ prevents its accumulation, thus diminishing the probability of enzyme inactivation. The most probable candidates responsible for H_2_O_2_ production are phenolic compounds in LF but their exact chemical nature remains to be studied. Further studies shall also reveal the relationships between the LPMO supporting efficiency of liquid streams and the method of pre-treatment and type of biomass. The results presented here may also provide a basis for development of LPMO-based methods for sensing H_2_O_2_ in complex redox-active environments.

The effect of LF on LPMO activity and on H_2_O_2_ levels may have a major effect on biomass conversion reactions, as illustrated by the studies of Müller et al. [76] on the enzymatic conversion of various types of biomasses with an LPMO-containing cellulase cocktail. For example, using H_2_O_2_ feeding under anaerobic conditions, Müller et al. found that the incorporation of fed H_2_O_2_ into LPMO products was almost stoichiometric when degrading Avicel, i.e., a relative clean substrate, whereas this stoichiometric relationship was not observed when using lignin-containing substrates [76]. Of note, these studies also led to the suggestion that current commercial cellulase cocktails may contain more LPMOs than needed to convert available H_2_O_2_ [76, 82], which is in accordance with the lack of enzyme concentration dependency found in the present study. Clearly, the interplay between biomass pretreatment and the efficiency of the subsequent enzymatic conversion process needs to be revisited in light of recent findings on LPMOs. It is important to note that phenolics and other soluble compounds in the LF, such as hemicellulose fragments, may inhibit the glycoside hydrolases in commercial enzyme cocktails and that the nature of pretreatment step, thus, may affect more than just LPMO functionality.

## Supplementary information

**Additional file 1: Table S1.** PCR primers used for screening of *T. reesei* transformants. **Fig. S1.** SDS-PAGE analysis of purified *Tr*LPMO9A. **Fig. S2.** Time curves of NAG_eq_ formation at different concentrations of *Sm*LPMO10A in reactions with LF pre-incubated at 25 °C. **Fig. S3.** Solid fraction (SF) from hydrothermal pretreatment of wheat straw supports the degradation of chitin (CNWs) by *Sm*LPMO10A. **Fig. S4.** Stoichiometry of the *Tr*LPMO9A reaction at 25 °C. **Fig. S5.** Time curves of Glc_eq_ formation at different concentrations of *Tr*LPMO9A, LF and the time period of pre-incubation of LF at 50 °C. **Fig. S6.** Dependence of the concentration of H_2_O_2_ ([H_2_O_2_]_*t*=0_), and the rate of its formation ($v_{{\left( {{\text{H}}_{2} {\text{O}}_{2} } \right)}}$) in the liquid fraction (LF) on the time period of pre-incubation of LF at 50 °C. **Fig. S7.** Measuring of [H_2_O_2_]_*t*=0_ after different times of pre-incubation of LF at 50 °C using *Sm*LPMO10A.

## Data Availability

All data generated during the present study are included in this published article.

## Abbreviations

AscA: ascorbic acid
BMCC: ^14^C-labeled bacterial microcrystalline cellulose
CNWs: ^14^C-labeled chitin nanowhiskers
EDTA: ethylenediaminetetraacetate
Glc_eq_: glucose equivalents
HRP: horseradish peroxidase
LF: liquid fraction from hydrothermal pretreatment of wheat straw
LPMO: lytic polysaccharide monooxygenase
NAG_eq_: *N*-acetylglucosamine equivalents

## References

[1] Pu Y, Hu F, Huang F, Davison BH, Ragauskas AJ (2013). Assessing the molecular structure basis for biomass recalcitrance during dilute acid and hydrothermal pretreatments. Biotechnol Biofuels.

[2] Cao G, Ximenes E, Nichols NN, Zhang L, Ladisch M (2013). Biological abatement of cellulase inhibitors. Bioresour Technol.

[3] Ko JK, Um Y, Park Y-C, Se J-H, Ki KH (2015). Compounds inhibiting the bioconversion of hydrothermally pretreated lignocellulose. Appl Microbiol Biotechnol.

[4] Zhai R, Hu J, Saddler JN (2016). What are the major components in steam pretreated lignocellulosic biomass that inhibit the efficacy of cellulase enzyme mixtures?. ACS Sustain Chem Eng..

[5] Vaaje-Kolstad G, Westereng B, Horn SJ, Liu Z, Zhai H, Sørlie M, Eijsink VGH (2010). An oxidative enzyme boosting the enzymatic conversion of recalcitrant polysaccharides. Science.

[6] Harris PV, Welner D, McFarland KC, Re E, Poulsen J-CN, Brown K, Salbo R, Ding H, Vlasenko E, Merino S, Xu F, Cherry J, Larsen S, Lo Leggio L (2010). Stimulation of lignocellulosic biomass hydrolysis by proteins of glycoside hydrolase family 61: structure and function of a large, enigmatic family. Biochemistry.

[7] Hu J, Arantes V, Pribowo A, Gourlay K, Saddler JN (2014). Substrate factors that influence the synergistic interaction of AA9 and cellulases during the enzymatic hydrolysis of biomass. Energy Environ Sci.

[8] Frommhagen M, Sforza S, Westphal AH, Visser J, Hinz SWA, Koetsier MJ, van Berkel WJH, Gruppen H, Kabel MA (2015). Discovery of the combined oxidative cleavage of plant xylan and cellulose by a new fungal polysaccharide monooxygenase. Biotechnol Biofuels.

[9] Martinez AT (2016). How to break down crystalline cellulose. Science.

[10] Crouch LI, Labourel A, Walton PH, Davies GJ, Gilbert HJ (2016). The contribution of non-catalytic carbohydrate binding modules to the activity of lytic polysaccharide monooxygenases. J Biol Chem.

[11] Bulakhov AG, Gusakov AV, Chekushina AV, Satrutdinov AD, Koshelev AV, Matys VY, Sinitsyn AP (2016). Isolation of homogeneous polysaccharide monooxygenases from fungal sources and investigation of their synergism with cellulases when acting on cellulose. Biochemistry.

[12] Chylenski P, Forsberg Z, Ståhlberg J, Varnai A, Lersch M, Bengtsson O, Sæbø S, Horn SJ, Eijsink VGH (2017). Development of minimal enzyme cocktails for hydrolysis of sulfite-pulped lignocellulosic biomass. J. Biotechnol..

[13] Chylenski P, Petrovic DM, Müller G, Dahlström M, Bengtsson O, Lersch M, Siika-aho M, Horn SJ, Eijsink VGH (2017). Enzymatic degradation of sulfite-pulped softwoods and the role of LPMOs. Biotechnol Biofuels.

[14] Ladeveze S, Haon M, Villares A, Cathala B, Grisel S, Herpoel-Gimbert I, Henrissat B, Berrin J-G (2017). The yeast *Geotrichum candidum* encodes functional lytic polysaccharide monooxygenases. Biotechnol Biofuels.

[15] Eibinger M, Ganner T, Bubner P, Rosker S, Kracher D, Haltrich D, Ludwig R, Plank H, Nidetzky B (2014). Cellulose surface degradation by a lytic polysaccharide monooxygenase and its effect on cellulase hydrolytic efficiency. J Biol Chem.

[16] Eibinger M, Sattelkow J, Ganner T, Plank H, Nidetzky B (2017). Single-molecule study of oxidative enzymatic deconstruction of cellulose. Nat. Commun..

[17] Pierce BC, Agger JW, Zhang Z, Wichmann J, Meyer AS (2017). A comparative study on the activity of fungal lytic polysaccharide monooxygenases for the depolymerization of cellulose in soybean spent flakes. Carbohydr Res.

[18] Du L, Ma L, Ma Q, Guo G, Han X, Xiao D (2018). Hydrolytic boosting of lignocellulosic biomass by a fungal lytic polysaccharide monooxygenase, *An*LPMO15g from *Aspergillus niger*. Ind Crop Prod..

[19] Du J, Song W, Zhang X, Zhao J, Liu J, Qu Y (2018). Differential reinforcement of enzymatic hydrolysis by adding chemicals and accessory proteins to high solid loading substrates with different pretreatments. Bioprocess Biosyst Eng..

[20] Sanhueza C, Carvajal G, Soto-Aguilar J, Lienqueo ME, Salazar O (2018). The effect of a lytic polysaccharide monooxygenase and a xylanase from *Gloephyllum trabeum* on the enzymatic hydrolysis of lignocellulosic residues using a commercial cellulase. Enzym Microb Technol..

[21] Couturier M, Ladeveze S, Sulzenbacher G, Ciano L, Fanuel M, Moreau C, Villares A, Cathala B, Chaspoul F, Frandsen KE, Labourel A, Herpöel-Gimbert I, Grisel S, Haon M, Lenfant N, Rogniaux H, Ropartz D, Davies GJ, Rosso M-N, Walton PH, Henrissat B, Berrin J-G (2018). Lytic xylan oxidases from wood-decay fungi unlock biomass degradation. Nat Chem Biol.

[22] Liu B, Krishnaswamyreddy S, Muraleedharan MN, Olson Å, Broberg A, Ståhlberg J, Sandgren M (2018). Side-by-side biochemical comparison of two lytic polysaccharide monooxygenases from the white-rot fungus *Heterobasidion irregulare* on their activity against crystalline cellulose and glucomannan. PLoS ONE.

[23] Hu J, Tian D, Renneckar S, Saddler JN (2018). Enzyme mediated nanofibrillation of cellulose by the synergistic actions of an endoglucanase, lytic polysaccharide monooxygenase (LPMO) and xylanase. Sci. Rep..

[24] Johansen KS (2016). Discovery and industrial applications of lytic polysaccharide mono-oxygenases. Biochem Soc Trans.

[25] Quinlan RJ, Sweeney MD, Lo Leggio L, Otten H, Poulsen J-CN, Johansen KS, Krogh KBRM, Jørgensen CI, Tovborg M, Anthonsen A, Tryfona T, Walter CP, Dupree P, Xu F, Davies GJ, Walton PH (2011). Insights into the oxidative degradation of cellulose by a copper metalloenzyme that exploits biomass components. Proc Natl Acad Sci USA.

[26] Phillips CM, Beeson WT, Cate JH, Marletta MA (2011). Cellobiose dehydrogenase and a copper-dependent polysaccharide monooxygenase potentiate cellulose degradation by *Neurospora crassa*. ACS Chem Biol.

[27] Meier KM, Jones SM, Kaper T, Hansson H, Koetsier MJ, Karkehabadi S, Solomon EI, Sandgren M, Kelemen B (2018). Oxygen activation by Cu LPMOs in recalcitrant carbohydrate polysaccharide conversion to monomer sugars. Chem Rev.

[28] Walton PH, Davies GJ (2016). On the catalytic mechanisms of lytic polysaccharide monooxygenases. Curr Opin Chem Biol.

[29] Frommhagen M, Westphal AH, van Berkel WJH, Kabel MA (2018). Distinct substrate specificities and electron-donating systems of fungal lytic polysaccharide monooxygenases. Front Microbiol..

[30] Kracher D, Scheiblbrandner S, Felice AKG, Breslmayr E, Preims M, Ludwicka K, Haltrich D, Eijsink VGH, Ludwig R (2016). Extracellular electron transfer systems fuel cellulose oxidative degradation. Science.

[31] Frommhagen M, Koetsier MJ, Westphal AH, Visser J, Hinz SWA, Vincken J-P, van Berkel WJH, Kabel MA, Gruppen H (2016). Lytic polysaccharide monooxygenases from *Myceliophthora thermophila* C1 differ in substrate preference and reducing agent specificity. Biotechnol Biofuels.

[32] Frommhagen M, Mutte SK, Westphal AH, Koetsier MJ, Hinz SWA, Visser J, Vincken J-P, Weijers D, van Berkel WJH, Gruppen H, Kabel MA (2017). Boosting LPMO-driven lignocelluloses degradation by polyphenol oxidase-activated lignin building blocks. Biotechnol Biofuels.

[33] Frommhagen M, Westphal AH, Hilgers R, Koetsier MJ, Hinz SWA, Visser J, Gruppen H, van Berkel WJH, Kabel MA (2018). Quantification of the catalytic performance of C1-cellulose-specific lytic polysaccharide monooxygenases. Appl Microbiol Biotechnol.

[34] Brenelli L, Squina F, Felby C, Cannella D (2018). Laccase-derived lignin compounds boost cellulose oxidative enzymes AA9. Biotechnol Biofuels.

[35] Hegnar OA, Petrovic DM, Bissaro B, Alfredsen G, Varnai A, Eijsink VGH (2019). Characterization of a lytic polysaccharide monooxygenase from *Gloephyllum trabeum* shows a pH-dependent relationship between catalytic activity and hydrogen peroxide production. Appl Environ Microbiol.

[36] Rodriguez-Zuniga UF, Cannella D, Giordano RC, Giordano RLC, Jørgensen H, Felby C (2015). Lignocellulose pretreatment technologies affect the level of enzymatic cellulose oxidation by LPMO. Green Chem.

[37] Westereng B, Cannella D, Agger JW, Jørgensen H, Andersen ML, Eijsink VGH, Felby C (2015). Enzymatic cellulose oxidation is linked to lignin by long-range electron transfer. Sci Rep..

[38] Muraleedharan MN, Zouraris D, Karantonis A, Topakas E, Sandgren M, Rova U, Christakopoulos P, Karnaouri A (2018). Effect of lignin fractions isolated from different biomass sources on cellulose oxidation by fungal lytic polysaccharide monooxygenases. Biotechnol Biofuels.

[39] Du B, Sharma LN, Becker C, Chen S-F, Mowery RA, van Walsum GP, Chambliss CK (2010). Effect of varying feedstock-pretreatment chemistry combinations on the formation and accumulation of potentially inhibitory degradation products in biomass hydrolysates. Biotechnol Bioeng.

[40] Rajan K, Carrier DJ (2014). Characterization of rice straw prehydrolyzates and their effect on the hydrolysis of model substrates using commercial *endo*-cellulase, β-glucosidase and cellulase cocktail. ACS Sustain Chem Eng..

[41] Zhao J, Chen H (2014). Stimulation of cellulases by small phenolic compounds in pretreated stover. J Agric Food Chem.

[42] Rasmussen H, Sørensen HR, Tanner D, Meyer AS (2017). New pentose dimers with bicyclic moieties from pretreated biomass. RSC Adv..

[43] Rasmussen H, Tanner D, Sørensen HR, Meyer AS (2017). New degradation compounds from lignocellulosic biomass pretreatment: routes for formation of potent oligophenolic enzyme inhibitors. Green Chem.

[44] Wu J, Collins SRA, Elliston A, Wellner N, Dicks J, Roberts IN, Waldron KW (2018). Release of cell wall phenolic esters during hydrothermal pretreatment of rice husk and rice straw. Biotechnol Biofuels.

[45] Zhai R, Hu J, Saddler JN (2018). Extent of enzyme inhibition by phenolics derived from pretreated biomass is significantly influenced by the size and carbonyl group content of phenolics. ACS Sustain Chem Eng..

[46] Bissaro B, Røhr ÅK, Skaugen M, Forsberg Z, Horn SJ, Vaaje-Kolstad G, Eijsink VGH (2017). Oxidative cleavage of polysaccharides by monocopper enzymes depends on H_2_O_2_. Nat Chem Biol.

[47] Bissaro B, Varnai A, Røhr ÅK, Eijsink VGH (2018). Oxidoreductases and reactive oxygen species in conversion of lignocellulosic biomass. Microbiol Mol Biol Rev.

[48] Kuusk S, Bissaro B, Kuusk P, Forsberg Z, Eijsink VGH, Sørlie M, Väljamäe P (2018). Kinetics of H_2_O_2_-driven degradation of chitin by a bacterial lytic polysaccharide monooxygenase. J Biol Chem.

[49] Hangasky JA, Iavarone AT, Marletta MA (2018). Reactivity of O_2_ versus H_2_O_2_ with polysaccharide monooxygenases. Proc Natl Acad Sci USA.

[50] Kuusk S, Kont R, Kuusk P, Heering A, Sørlie M, Bissaro B, Eijsink VGH, Väljamäe P (2019). Kinetic insights into the role of the reductant in H_2_O_2_-driven degradation of chitin by a bacterial lytic polysaccharide monooxygenase. J Biol Chem.

[51] Eijsink VGH, Petrovic D, Forsberg Z, Mekasha S, Røhr ÅK, Varnai A, Bissaro B, Vaaje-Kolstad G (2019). On the functional characterization of lytic polysaccharide monooxygenases (LPMOs). Biotechnol Biofuels.

[52] Kuusk S, Sørlie M, Väljamäe P (2015). The predominant molecular state of bound enzyme determines the strength and type of product inhibition in the hydrolysis of recalcitrant polysaccharides by processive enzymes. J Biol Chem.

[53] Velleste R, Teugjas H, Väljamäe P (2010). Reducing end-specific fluorescence labeled celluloses for cellulase mode of action. Cellulose.

[54] Jalak J, Kurašin M, Teugjas H, Väljamäe P (2012). Endo-exo synergism in cellulose hydrolysis revisited. J Biol Chem.

[55] Vaaje-Kolstad G, Houston DR, Riemen AHK, Eijsink VGH, van Aalten DMF (2005). Crystal structure and binding properties of the *Serratia marcescens* chitin-binding protein CBP21. J Biol Chem.

[56] Colot HV, Park G, Turner GE, Ringelberg C, Crew CM, Litvinkova L, Weiss RL, Borkovich KA, Dunlap JC (2006). A high-throughput gene knockout procedure for *Neurospora* reveals functions for multiple transcription factors. Proc Natl Acad Sci USA.

[57] Colabardini AC, Valkonen M, Huuskonen A, Siika-Aho M, Koivula A, Goldman GH, Saloheimo M (2016). Expression of two novel β-glucosidases from *Chaetomium atrobrunneum* in *Trichoderma reesei* and characterization of the heterologous protein products. Mol Biotechnol.

[58] Mach RL, Schindler M, Kubicek CP (1994). Transformation of *Trichoderma reesei* based on hygromycin B resistance using homologous expression signals. Curr Genet.

[59] Landowski CP, Huuskonen A, Wahl R, Westerholm-Parvinen A, Kanerva A, Hänninen AL, Salovuori N, Penttilä M, Natunen J, Ostermeier C, Helk B, Saarinen J, Saloheimo M (2015). Enabling low cost biopharmaceuticals: a systematic approach to delete proteases from a well-known protein production host *Trichoderma reesei*. PLoS ONE.

[60] Bailey MJ, Tähtiharju J (2003). Efficient cellulase production by *Trichoderma reesei* in continuous cultivation on lactose medium with a computer-controlled feeding strategy. Appl Microbiol Biotechnol.

[61] Bailey M, Nevalainen H (1981). Induction, isolation and testing of stable *Trichoderma reesei* mutants with improved production of solubiling cellulase. Enzym Microb Technol.

[62] Bailey MJ, Biely P, Poutanen K (1992). Interlaboratory testing of methods for assay of xylanase activity. J Biotechnol.

[63] Stålbrand H, Siika-aho M, Tenkanen M, Viikari L (1993). Purification and characterization of two beta-mannanases from *Trichoderma reesei*. J Biotechnol.

[64] Goldschmid O, Sarkanen KV, Ludwig CH (1971). Ultraviolet spectra. Lignins: occurrence, formation, structure and reactions.

[65] Sluiter JB, Ruiz RO, Scarlata CJ, Sluiter AD, Templeton DW (2010). Compositional analysis of lignocellulosic feedstocks. 1. Review and description of methods. J Agric Food Chem..

[66] Tanghe M, Danneels B, Camattari A, Glieder A, Vandenberghe I, Devreese B, Stals I, Desmet T (2015). Recombinant expression of *Trichoderma reesei* Cel61A in *Pichia pastoris*: optimizing yield and N-terminal processing. Mol Biotechnol.

[67] Guo Z-P, Duquesne S, Bozonnet S, Nicaud J-M, Marty A, O’Donohue MJ (2017). Expressing accessory proteins in cellulolytic *Yarrowia lipolytica* to improve the conversion yield of recalcitrant cellulose. Biotechnol Biofuels.

[68] Hansson H, Karkehabadi S, Mikkelsen N, Douglas NR, Kim S, Lam A, Kaper T, Kelemen B, Meier KK, Jones SM, Solomon EI, Sandgren M (2017). High-resolution structure of lytic polysaccharide monooxygenase from *Hypocrea jecorina* reveals a predicted linker as an integral part of the catalytic domain. J Biol Chem.

[69] Song B, Li B, Wang X, Shen W, Park S, Collings C, Feng A, Smith SJ, Walton JD, Ding S-Y (2018). Real-time imaging reveals that lytic polysaccharide monooxygenase promotes cellulase activity by increasing cellulose accessibility. Biotechnol Biofuels.

[70] Kari J, Andersen M, Borch K, Westh P (2017). An inverse Michaelis-Menten approach for interfacial enzyme kinetics. ACS Catal..

[71] Cannella D, Möllers KB, Frigaard N-U, Jensen PE, Bjerrum MJ, Johansen KS, Felby C (2016). Light-driven oxidation of polysaccharides by photosynthetic pigments and a metalloenzyme. Nat Commun..

[72] Möllers KB, Mikkelsen H, Simonsen TI, Cannella D, Johansen KS, Bjerrum MJ, Felby C (2017). On the formation and role of reactive oxygen species in light-driven LPMO oxidation of phosphoric acid swollen cellulose. Carbohydr Res.

[73] Kittl R, Kracher D, Burgstaller D, Haltrich D, Ludwig R (2012). Production of four *Neurospora crassa* lytic polysaccharide monooxygenases in *Pichia pastoris* monitored by a fluorimetric assay. Biotechnol Biofuels.

[74] Danneels B, Tanghe M, Desmet T (2019). Structural features on the substrate-binding surface of fungal lytic polysaccharide monooxygenases determine their oxidative regioselectivity. Biotechnol J.

[75] Sroka Z, Cisowski W (2003). Hydrogen peroxide scavenging, antioxidant and anti-radical activity of some phenolic acids. Food Chem Toxicol.

[76] Müller G, Chylenski P, Bissaro B, Eijsink VGH, Horn SJ (2018). The impact of hydrogen peroxide supply on LPMO activity and overall saccharification efficiency of a commercial cellulase cocktail. Biotechnol Biofuels.

[77] Le DM, Sørensen HR, Knudsen NO, Schjoerring JK, Meyer AS (2014). Biorefining of wheat straw: accounting for the distribution of mineral elements in pretreated biomass by an extended pretreatment-severity equation. Biotechnol Biofuels.

[78] Kont R, Kurašin M, Teugjas H, Väljamäe P (2013). Strong cellulase inhibitors from hydrothermal pretreatment of wheat straw. Biotechnol Biofuels.

[79] Rajan K, Carrier DJ (2016). Insights into *exo*-cellulase inhibition by the hot water hydrolyzates of rice straw. ACS Sustainable Chem. Eng..

[80] Zhai R, Hu J, Saddler JN (2018). Understanding the slowdown of whole slurry hydrolysis of steam pretreated lignocellulosic woody biomass catalyzed by an up-to-date enzyme cocktail. Sustain Energy Fuels.

[81] Peciulyte A, Samuelsson L, Olsson L, McFarland KC, Frickmann J, Østergård L, Halvorsen R, Scott BR, Johansen KS (2018). Redox processes acidify and decarboxylate steam-pretreated lignocellulosic biomass and are modulated by LPMO and catalase. Biotechnol Biofuels.

[82] Chylenski P, Bissaro B, Sørlie M, Røhr ÅK, Varnai A, Horn SJ, Eijsink VGH (2019). Lytic polysaccharide monooxygenases in enzymatic processing of lignocellulosic biomass. ACS Catal..

