# Supplementary material for: The liquid fraction from hydrothermal pretreatment of wheat straw provides lytic polysaccharide monooxygenases with both electrons and H2O2 co-substrate
Source: Biotechnol Biofuels. 2019 Oct 8;12:235. doi: 10.1186/s13068-019-1578-5 (PMC6781412; doi:10.1186/s13068-019-1578-5)
Supplement: Supplementary file 1 — Additional file 1: Table S1. PCR primers used for screening of T. reesei transformants. Fig. S1. SDS-PAGE analysis of purified TrLPMO9A. Fig. S2. Time curves of NAGeq formation at different concentrations of SmLPMO10A in reactions with LF pre-incubated at 25 °C. Fig. S3. Solid fraction (SF) from hydrothermal pretreatment of wheat straw supports the degradation of chitin (CNWs) by SmLPMO10A. Fig. S4. Stoichiometry of the TrLPMO9A reaction at 25 °C. Fig. S5. Time curves of Glceq formation at different concentrations of TrLPMO9A, LF and the time period of pre-incubation of LF at 50 °C. Fig. S6. Dependence of the concentration of H2O2 ([H2O2]t=0), and the rate of its formation ($v_{{\left( {{\text{H}}_{2} {\text{O}}_{2} } \right)}}$vH2O2) in the liquid fraction (LF) on the time period of pre-incubation of LF at 50 °C. Fig. S7. Measuring of [H2O2]t=0 after different times of pre-incubation of LF at 50 °C using SmLPMO10A. [file 13068_2019_1578_MOESM1_ESM.docx]

Additional file 1:

**The liquid fraction from hydrothermal pretreatment of wheat straw provides lytic polysaccharide monooxygenases with both electrons and H_2_O_2_ co-substrate**

**Table S1. PCR primers used for screening of *T. reesei* transformants**

|  | 5’ primer | 3’ primer |
| --- | --- | --- |
| *cbh1* 5’ integration | TO95 GCTGTTCCTACAGCTCTTTC | SO3 CCAGTAGCCACCGCCAGTGC |
| *cbh1* 3’ integration | TO47 CCTATGAGTCGTTTACCCAGA | TO08 GGTTGACTTACTCCAGATCG |
| *cbh1* ORF | T1720 CCTGACGCTATCTTCTTGTTGG | T1721  CGCGCATGTTTGTCCATCAAAC |


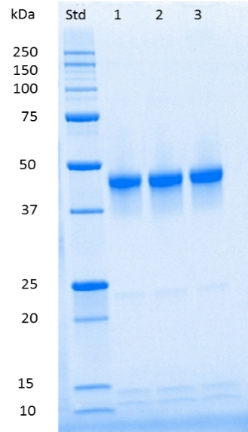


**Fig. S1. SDS-PAGE analysis of purified *Tr*LPMO9A.** SDS-PAGE analysis was performed using 4-20 % Criterion Stain Free precast gel and BioRad Stain Free Imaging System (BioRad, California, USA). Purified *Tr*LPMO9A is on lines 1-3. “Std” denotes molecular weight standard.


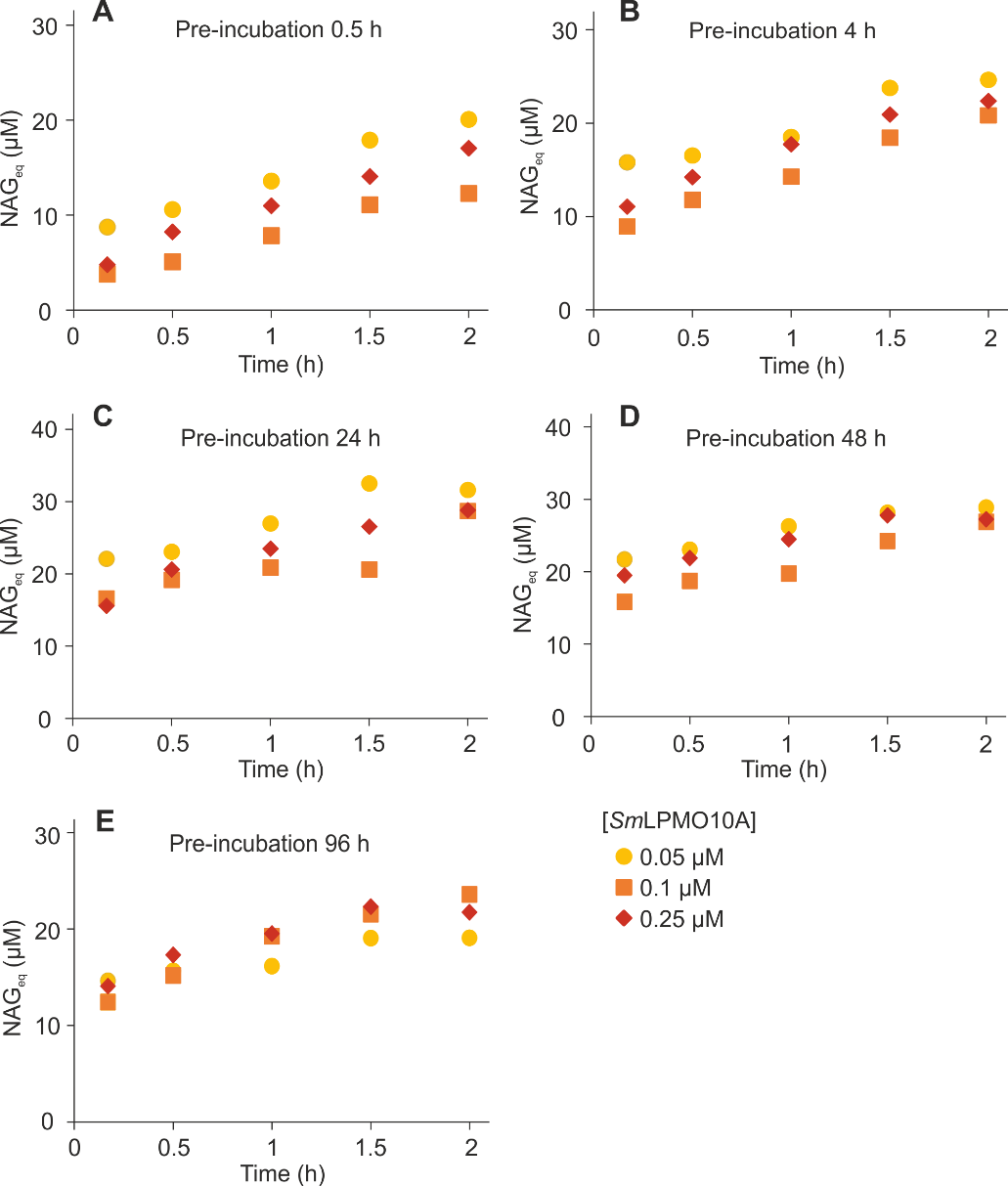


**Fig. S2. Time curves of NAG_eq_ formation at different concentrations of *Sm*LPMO10A in reactions with LF pre-incubated at 25 ºC.** Reactions were made in 50 mM sodium acetate (pH 5.0) at 25 ºC. The concentration of CNWs was 1.0 g L^-1^ and that of LF was 10% (v/v). The concentration of *Sm*LPMO10A was 0.05 µM, 0.1 µM, or 0.25 µM (as defined in the plot). Before setting up the reactions the LF was pre-incubated at 25 ºC in aerobic conditions for (A) 0.5 h, (B) 4 h, (C) 24 h, (D) 48 h, or (E) 96 h.


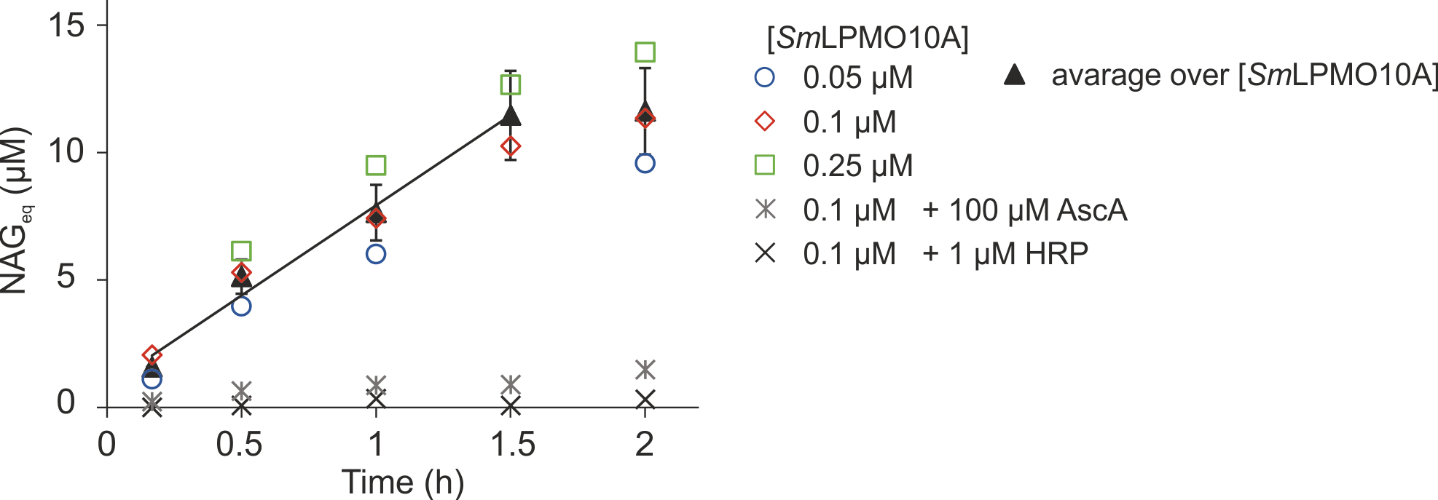


**Fig. S3. Solid fraction (SF) from hydrothermal pretreatment of wheat straw supports the degradation of chitin (CNWs) by *Sm*LPMO10A.** Reactions were made in 50 mM sodium acetate (pH 5.0) at 25 ºC. The concentration of CNWs was 1.0 g L^-1^ and that of *Sm*LPMO10A was 0.05 µM, 0.1 µM, or 0.25 µM (as defined in the plot). The concentration of SF was 10 g L^-1^ and before use in the LPMO experiment, the SF was pre-incubated at 25 ºC overnight. Control experiments show the results obtained with 0.1 µM *Sm*LPMO10A but in the presence of 1 µM horseradish peroxidase. In another control experiment the concentration of *Sm*LPMO10A was 0.1 µM but the SF was replaced with an equivalent amount (on a volume basis) of the supernatant obtained after centrifugation of the SF stock suspension. In this experiment ascorbic acid was added (to 100 µM) to ensure efficient priming reduction. Solid line represent linear regression of the data (up to the 1.5 h time point).


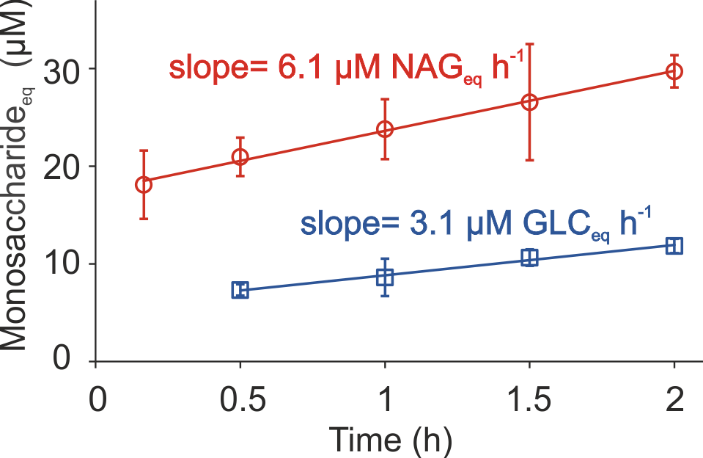


**Fig. S4. Stoichiometry of the *Tr*LPMO9A reaction at 25 ºC.** Reactions were made in 50 mM sodium acetate (pH 5.0) at 25 ºC. The concentration of LF was 10% (v/v), and, before use in the LPMO reaction, the LF was pre-incubated at 25 ºC for 24 h. In the experiments with chitin (red circles), the concentration of CNWs was 1.0 g L^-1^ and that of *Sm*LPMO10A was 0.05 µM, 0.1 µM, or 0.25 µM. In the experiments with cellulose (blue squares), the concentration of BMCC was 0.6 g L^-1^ and that of *Tr*LPMO9A was 0.1 µM, 0.2 µM, or 0.5 µM. Time curves show the formation of soluble products expressed in NAG_eq_ (for the experiments with chitin) or Glc_eq_ (for the experiments with cellulose). Error bars represent S.D. and are from three independent measurements each made in single parallel but at different concentration of LPMO (product formation was essentially independent of the LPMO concentration). Solid lines show linear regression of the data. The rate of H_2_O_2_ formation (1.5 ± 0.2 µM h^-1^) was calculated from the rate of the *Sm*LPMO10A reaction (6.1 ± 0.7 µM NAG_eq_ h^-1^) and a previously determined stoichiometry of 4 NAG_eq_/H_2_O_2_ (Kuusk et al. 2018). The stoichiometry of the *Tr*LPMO9A reaction (2.1 ± 0.3 Glc_eq_/H_2_O_2_) was calculated from the rate of *Tr*LPMO9A reaction (3.1 ± 0.3 µM NAG_eq_ h^-1^) and the rate of H_2_O_2_ formation (1.5 ± 0.2 µM h^-1^).


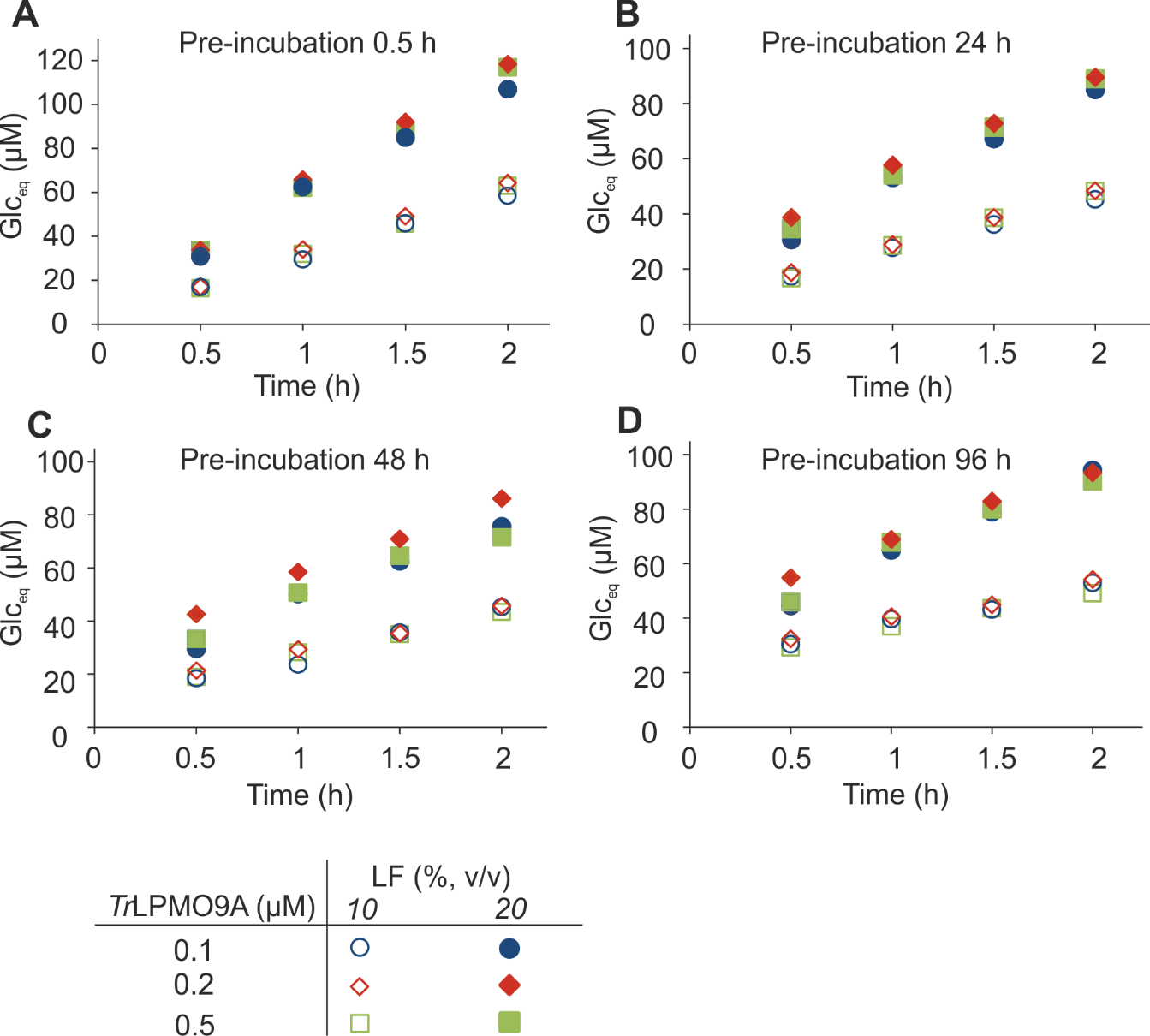


**Fig. S5. Time curves of Glc_eq_ formation at different concentrations of *Tr*LPMO9A, LF and the time period of pre-incubation of LF at 50 ºC.** Reactions were made in 50 mM sodium acetate (pH 5.0) at 50 ºC. The concentration of BMCC was 0.6 g L^-1^ and that of LF was 10% or 20% (v/v) as indicated in the plot. The concentration of *Tr*LPMO9A was 0.1 µM, 0.2 µM, or 0.5 µM (as indicated in the plot). Before starting the *Tr*LPMO9A reaction the LF was pre-incubated at 50 ºC in aerobic conditions for (A) 0.5 h, (B) 24 h, (C) 48 h, or (D) 96 h.


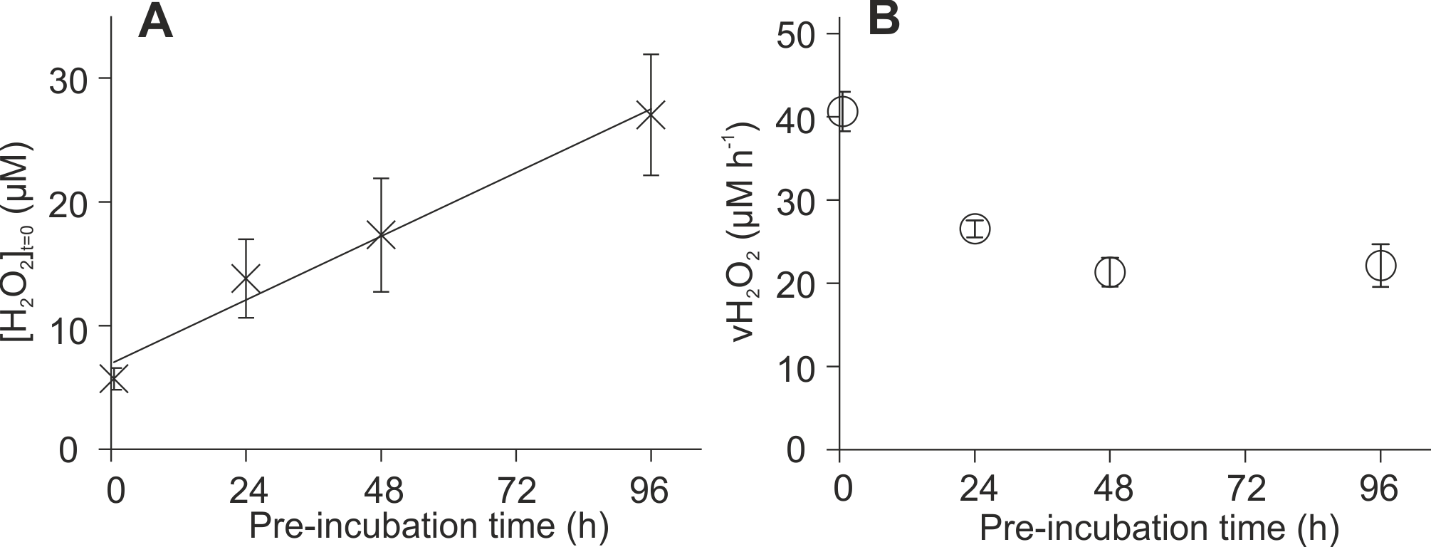


**Fig. S6. Dependence of the concentration of H_2_O_2_ ([H_2_O_2_]_t=0_), and the rate of its formation (*v*_H2O2_) in the liquid fraction (LF) on the time period of pre-incubation of LF at 50 ºC.** Reactions were made in 50 mM sodium acetate (pH 5.0) at 50 ºC. The concentration of BMCC was 0.6 g L^-1^ and that of LF was 20% (v/v) (data for 10 % (v/v) LF appear in Fig. 5 of the main paper). The panels show the dependence of (A) the [H_2_O_2_]_t=0_ and (B) the *v*_H2O2_, on the pre-incubation time of LF at 50 ºC. The values of [H_2_O_2_]_t=0_ and *v*_H2O2_ were found by fitting rates derived from Fig. S5 to Eq 2 by linear regression, using a stoichiometry (*n*) of 1.32 Glc_eq_/H_2_O_2_. Error bars represent S.D. and are from three independent measurements each made in single parallel but at different concentration of *Tr*LPMO9A (Fig. S5).


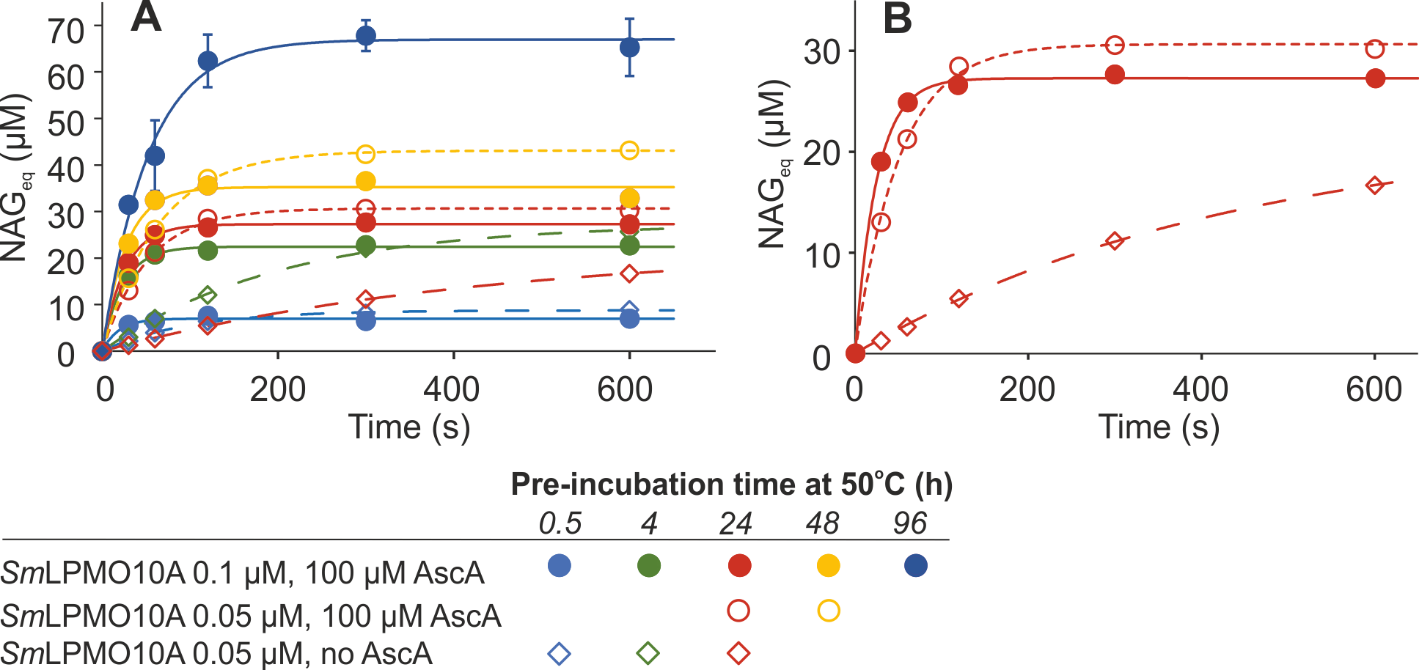


**Fig. S7. Measuring of [H_2_O_2_]_t=0_ after different times of pre-incubation of LF at 50 ºC using *Sm*LPMO10A.** *Sm*LPMO10A reactions were made in 50 mM sodium acetate (pH 5.0) at 25 ºC. The concentration of CNWs was 1.0 g L^-1^ and that of LF was 10% (v/v). The concentration of *Sm*LPMO10A was 0.05 µM or 0.1 µM. Where indicated, the reactions were supplied with 100 µM ascorbic acid to ensure efficient priming reduction. Before starting the *Sm*LPMO10A reaction, the LF was pre-incubated at 50 ºC for the time period indicated in the plot. Solid lines show best-fits of non-linear regression analysis according to Eq 3. The values of [H_2_O_2_]_t=0_ were found from the *n*[H_2_O_2_]_t=0_ values obtained from the non-linear regression analysis using the value of *n* = 4 NAG_eq_/H_2_O_2_. Panel A shows all time curves. For clarity, the data obtained with LF that was pre-incubated for 24 h are also shown in panel B. Two conclusions can be drawn from the data in panel B. (i) The efficiency of the priming reduction decreases with pre-incubation of LF at 50 ºC as evidenced by the slower reaction in the absence of added ascorbic acid (see Kuusk et al 2019). (ii) Increasing the *Sm*LPMO10A concentration increases the initial rate of NAG_eq_ formation.

**Additional references**

Kuusk S, Bissaro B, Kuusk P, Forsberg Z, Eijsink VGH, Sørlie M, Väljamäe P. Kinetics of H_2_O_2_-driven degradation of chitin by a bacterial lytic polysaccharide monooxygenase. J. Biol. Chem. 2018;293:523-31.

S. Kuusk, R. Kont, P. Kuusk, A. Heering, M. Sørlie, B. Bissaro, V. G. H. Eijsink and P. Väljamäe, *J. Biol. Chem.*, 2019, **294**, 1516-1528.
